# Positionspapier für die standardisierte Anwendung von Transjugulärem Intrahepatischem Portosystemischem Shunt (TIPS) bei Erwachsenen

**DOI:** 10.1055/a-2675-4735

**Published:** 2025-11-10

**Authors:** Jonel Trebicka, Christian Steib, Alexander Zipprich, Cristina Ripoll, Johannes Kluwe, Michael Schultheiß, Andreas A. Schnitzbauer, Bernhard Meyer, Christian Jansen, Hauke Heinzow, Philipp Papprottka, Carsten Meyer, Max Seidensticker, Harald Ittrich, Frank Erhard Uschner, Wenyi Gu, Niklas Aehling, Ulf Neumann, Karel Caca, Michael Köhler, Leon Louis Seifert, Dominik Betinger, Tony Bruns, Matthias Dollinger, Michael Praktiknjo, Thomas Berg, Moritz Wildgruber, Michael B. Pitton, Holger Goessmann, Alexander Gerbes, Martin Rössle, Freigegeben durch:, Heiner Wedemeyer, Ulrike Denzer, Petra Lynen

**Affiliations:** 1 Medizinische Klinik B (Gastroenterologie, Hepatologie, Endokrinologie, Klinische Infektiologie), Universitätsklinikum Münster, Münster; 2 Medizinische Klinik und Poliklinik II, LMU Klinikum München; 3 Innere Medizin IV (Gastroenterologie, Hepatologie, Infektiologie, interdisziplinäre Endoskopie), Universitätsklinikum Jena, FSU Jena, Jena; 4 Medizinische Klinik und Poliklinik, Universitätsklinikum Hamburg-Eppendorf, Hamburg; 5 Klinik für Innere Medizin II, Universitätsklinikum Freiburg, Medizinische Fakultät, Albert-Ludwigs-Universität Freiburg, Deutschland; 6 Klinik für Chirurgie, Universitätsklinikum Regensburg, Regensburg; 7 Klinik für Diagnostische und Interventionelle Radiologie, Medizinische Hochschule Hannover, Hannover; 8 Medizinische Klinik I, Universitätsklinikum Bonn, Bonn; 9 Medizinische Klinik I, Krankenhaus der Barmherzigen Brüder, Trier, Abteilung für Interventionelle Radiologie, Klinikum rechts der Isar der Technischen Universität München, München; 10 Abteilung für Interventionelle Radiologie, Klinikum rechts der Isar der Technischen Universität München, München; 11 Klinik für Diagnostische und Interventionelle Radiologie Universitätsklinikum Bonn; 12 Klinik und Poliklinik für Radiologie, Universitätsklinikum, LMU Klinikum, München; 13 Abteilung für Diagnostische und Interventionelle Radiologie, Schön Klinik Hamburg SE & KO. KG, Hamburg; 14 Klinikum Landshut; 15 Bereich Hepatologie, Klinik und Poliklinik für Onkologie, Gastroenterologie, Hepatologie, und Pneumologie, Universitätsklinikum Leipzig; 16 Institut für Klinische Radiologie, Medizinische Fakultät – Universität Münster – und Universitätsklinikum Münster, Münster; 17 Klinik für Diagnostische und Interventionelle Radiologie, Universitätsklinikum Mainz; 18 Klinik für Radiologie, Universitätsklinikum Essen, Essen; 19 Medizinische Klinik III, RWTH Universitätsklinikum Aachen, Aachen; 20 Internistisches Klinikum München Süd, Klinik für Gastroenterologie und Innere Medizin, München

**Keywords:** Leber, TIPS, Leberzirrhose, Portale Hypertension, Liver, TIPS, Liver Cirrhosis, Portal Hypertension

## Abstract

Portale Hypertonie ist mit erheblicher Morbidität und Mortalität verbunden. Die Leberzirrhose macht bis zu 90 % der Fälle von portaler Hypertonie aus, während etwa 10 % auf nicht-zirrhotische Faktoren, einschließlich vaskulärer Lebererkrankungen, zurückzuführen sind.

Diese Erkrankung kann zu schweren Komplikationen führen, wie der Entwicklung von gastroösophagealen Varizen, die das Risiko von varikösen Blutungen erheblich erhöhen. Weitere häufige Komplikationen der portalen Hypertonie sind Aszites und hepatorenales Syndrom (HRS).

Eine transjuguläre intrahepatische portosystemische Shunt (TIPS)-Implantation wird als die effektivste Behandlung zur Bewältigung der portalen Hypertonie angesehen. Studien zeigen, dass die TIPS-Implantation die Überlebensraten bei Patienten mit wiederkehrendem Aszites sowie bei ausgewählten Patienten mit refraktärem Aszites und varikösen Blutungen verbessern kann. Allerdings können periinterventionelle und postinterventionelle Komplikationen die Anwendung von TIPS einschränken. Neueste Entwicklungen bei Geräten, Techniken und prophylaktischen Medikamenten zielen darauf ab, das Risiko von Komplikationen nach dem Eingriff zu minimieren.

Dieses interdisziplinäre Positionspapier fasst Empfehlungen und Anleitung zur Patientenwahl, zu Indikationen und Kontraindikationen, zu Techniken sowie zur Nachsorge von Patienten zusammen, die in Deutschland ein TIPS-Verfahren erhalten.

## Abstract (en)

**Abstract:**

Portal hypertension is associated with significant morbidity and mortality. Liver cirrhosis accounts for up to 90 % of portal hypertension cases, while approximately 10 % are due to non-cirrhotic factors, including vascular liver diseases.

This condition can lead to various complications, such as the development of gastroesophageal varices, which greatly increase the risk of variceal bleeding. Other common complications of portal hypertension include ascites and hepatorenal syndrome (HRS).

The transjugular intrahepatic portosystemic shunt (TIPS) procedure is regarded as the most effective treatment for managing portal hypertension. Research shows that TIPS insertion can improve survival rates for patients with recurrent ascites, as well as for select individuals with refractory ascites and variceal bleeding. However, peri-interventional and post-interventional complications may pose challenges to the use of TIPS. Recent advancements in devices, techniques, and prophylactic medications aim to minimize the risk of complications following this procedure.

This interdisciplinary position paper summarizes recommendations and guidance regarding patient selection, indications and contraindications, techniques, and follow-up care for patients undergoing TIPS in Germany.

## Indikationsstellung zum TIPS

### TIPS-Anlage bei Aszites und bei Nierenfunktionsstörung

Grundsätzlich ist bei jedem Patienten mit refraktärem oder rezidivierendem Aszites die Indikation zur TIPS-Anlage zu prüfen und eine Lebertransplantation als die kurative Therapie zu erwägen. Als rezidivierend wird ein Aszites definiert, wenn trotz maximaler verträglicher diuretischer und medikamentöser Therapie mehr als ≥ 3 großvolumige (> 4 L) Parazentesen innerhalb eines Jahres erforderlich werden, oder aber mindestens 2 großvolumige Parazentesen innerhalb von 3 Wochen 1 2 . Die klassischen Refraktäritätskriterien des Internationalen Aszitesclubs (ICA) von 1996 3 sind unter anderem fehlendes Ansprechen auf maximale Diuretikadosen, Diuretika-induzierte Komplikationen (und die Maximaldosis nicht erreicht werden kann), frühes Wiederauftreten von Aszites innerhalb von vier Wochen nach Flüssigkeitsmobilisierung, persistierender Aszites trotz Natriumrestriktion. Diese Kriterien sind im klinischen Alltag schwer umzusetzen und sollten aus heutiger Perspektive aufgrund der damit verbundenen Nierenfunktionseinschränkung nicht mehr, oder nur noch in Ausnahmefällen, eingesetzt werden 4 .

Die TIPS-Anlage führt in ca. 60 % der Fälle zu einer Aszites-Kontrolle 5 6 7 8 9 . Positive zusätzliche Effekte des TIPS sind eine Verbesserung der Nierenfunktion, eine Reduktion der Inzidenz spontan-bakterieller Peritonitiden, sowie eine Verbesserung des Ernährungszustandes 10 11 12 . Der TIPS erhöht prinzipiell das Risiko der Entwicklung oder auch Verschlechterung einer vorbestehenden hepatischen Enzephalopathie (HE), allerdings seit der Verwendung gecoverter Stents mit 6–8 mm Stentdiametern (und Coiling von Kollateralen) ist die Frequenz der post-TIPS-HE deutlich rückläufig. Durch die TIPS-assoziierte Verbesserung der systemischen Hämodynamik, Nierenfunktion und des Ernährungsstatus ist auch eine Verbesserung der HE möglich, sodass vorangegangene HE-Episoden nicht grundsätzlich als Kontraindikation für eine TIPS-Anlage angesehen werden sollten 13 14 15 . Hinsichtlich des Überlebens bei Patienten mit therapierefraktärem oder rezidivierenden Aszites zeigen sowohl einzelne frühere Studien mit unbeschichteten Stents 16 17 , als auch spätere Studien mit beschichteten Stents 2 18 19 einen Vorteil für Patienten, die einen TIPS erhalten haben 20 21 .

Der hepatische Hydrothorax ist mit einer ungünstigen Prognose und mit einer mittleren Überlebensdauer zwischen 8 und 12 Monaten assoziiert. Die Kriterien zur Definition eines refraktären Hydrothorax sind bisher nicht einheitlich festgelegt. Es sollte jedoch analog zum refraktären Aszites definiert werden als ein Hydrothorax, der trotz optimal kontrollierter medizinischer Diuretikatherapie wiederholt eine Thorakozentese erforderlich macht. Wenn angezeigt und möglich ist eine Lebertransplantation die beste Behandlungsoption für Patienten mit refraktärem hepatischem Hydrothorax. Die meisten Daten zur TIPS-Anlage bei Patienten mit einem hepatischen Hydrothorax basieren auf kleinen retrospektiven Kohorten. In einer Metaanalyse von sechs retrospektiven Studien war die Wirksamkeit einer TIPS-Anlage bei der Kontrolle des hepatischen Hydrothorax in 56 % (95 % KI 45–67) vollständig und in 18 % (95 % KI 11–24) partiell. In einer Propensity-Score-gematchten Kohorte von 243 Patienten mit Leberzirrhose und hepatischem Hydrothorax war das kumulative Überleben nach TIPS vergleichbar mit dem von Patienten, die keinen TIPS erhalten hatten. Das Ansprechen auf eine TIPS-Anlage hängt wie bei einem therapierefraktären Aszites maßgeblich von der Leberfunktion ab 22 23 24 25 26 . Zusammenfassend ist der TIPS eine sinnvolle effektive Behandlung der portalen Hypertonie oder als Überbrückung bis zur Lebertransplantation.

Die Definitionen des hepatorenalen Syndroms (HRS) bei Patienten mit Leberzirrhose in die HRS-Typen 1 und 2 wurden in den letzten Jahren überarbeitet. Angesichts unterschiedlicher Pathophysiologien, die dem HRS Typ 1 und Typ 2 zugrunde liegen, wird nun empfohlen, die nephrologische KDIGO-Klassifikation (Kidney Disease Improving Global Outcomes) für das akute Nierenversagen (AKI) anzuwenden 27 28 . In dieser Einteilung ist das AKI durch eine relative Veränderung der GFR zum Ausgangswert und nicht durch einen fixierten Grenzwert definiert. Mit dieser neuen Einteilung fällt das klassische hepatorenale Syndrom Typ 1 unter die Rubrik AKI und das klassische hepatorenale Syndrom Typ 2 in den Bereich non-AKI (NAKI) oder chronic kidney disease (CKD). Die bisher veröffentlichten Studien zur Effektivität einer TIPS-Anlage bei Patienten mit AKI erfolgten jedoch mit der traditionellen HRS-Definition 29 .

Zwei retrospektive Studien untersuchten anhand von ICD-kodierten Daten den Einfluss einer TIPS-Anlage auf die Kurzzeitletalität bei Patienten mit HRS. Die Ergebnisse einer der Studien zeigten, dass die TIPS-Implantation mit einer Verringerung der Kurzzeitletalität assoziiert war [adjustierte OR 0,43 (95 % CI 0,30–0,62)]. Eine Unterscheidung zwischen HRS-AKI, NAKI oder CKD (bzw. klassischem HRS Typ 1 und 2) war aufgrund des Studiendesigns jedoch nicht möglich 30 . Die weitere Studie untersuchten mehr als 14 000 TIPS-Anlagen in Deutschland und konnten den positiven Überlebenseffekt bestätigen 31 .

Bei HRS-NAKI oder HRS-CKD (HRS-2) bewirkt der TIPS in der Regel eine deutliche Verbesserung der Nierenfunktion 14 29 32 . Eine frühe, signifikante und nachhaltige Verbesserung der Nierenfunktion nach TIPS-Implantation konnte kürzlich auch für Patienten mit HRS-CKD, unabhängig vom Ausgangsstadium der CKD, gezeigt werden. 11 . Randomisiert-kontrollierte Daten zur TIPS-Anlage bei Patienten mit HRS-AKI fehlen zum aktuellen Zeitpunkt, werden jedoch durch die aktuell rekrutierende Liver-Hero-Studie erwartet 33 .

### TIPS-Anlage bei portal-hypertensiver Blutung bei Leberzirrhose

#### Notfall-TIPS und präemptiver TIPS

Gastroösophageale Varizenblutungen sind bei Patienten mit Leberzirrhose mit einer jährlichen Inzidenz von 5–15 % zwar seltener als Aszites, stellen jedoch die Zweithäufigste Indikation zur TIPS Anlage dar 34 . Ihre Standardtherapie umfasst die Kombination aus endoskopischer Varizenbehandlung, vasoaktiven Medikamenten zur Pfortaderdrucksenkung und Antibiotikabehandlung 34 . Durch diese Maßnahmen konnte die Letalität der ÖV-Blutung in den letzten Jahrzehnten deutlich gesenkt werden 35 . Dennoch bleibt eine erhebliche Frühsterblichkeit von etwa 14–16 % bestehen, die insbesondere bei Patienten mit fortgeschrittener Zirrhose nach einer Ösophagusvarizenblutung zu verzeichnen ist 35 36 . Der Grund hierfür besteht darin, dass trotz optimaler endoskopischer und medikamentöser Therapie etwa 10–15 % der Blutungsereignisse nicht zu kontrollieren sind, oder innerhalb der ersten 5 Tage rezidivieren 37 . Bei Patienten, bei denen eine Varizenblutung endoskopisch nicht gestillt werden kann, stellt die Notfall-TIPS -Implantation eine effektive Maßnahme dar 38 39 40 41 . Obwohl diese bei mehr als 90 % der Fälle gelingt, bleibt die Frühletalität durch Multiorganversagen oder Sepsis hoch 42 43 und liegt innerhalb der ersten 4–8 Wochen bei etwa 35–55 % 38 39 40 41 . Deshalb sollte im Einzelfall bei unkontrollierter Sepsis oder Leberversagen (Bilirubin > 10 mg/dl) die Indikation und der Zeitpunkt kritisch diskutiert werden.

Die trotz Blutstillung in der Notfallendoskopie bestehende hohe Frühletalität durch die Rezidivblutung stellt die Rationale für ein proaktiveres Vorgehen dar und begründet das Konzept des präemptiven TIPS , also der frühzeitigen (innerhalb der ersten 72 Stunden) TIPS-Implantation nach primär endoskopisch-medikamentöser Blutungsstillung. Drei randomisierte, kontrollierte Studien (RCT) unterstützen dieses Konzept und zeigen einen Überlebensvorteil bei ausgewählten Patienten 44 45 . Risikopatienten mit Child-Pugh B (≥ 8) und aktiver Blutung bei der initialen Endoskopie oder Child-Pugh C (< 14) haben eine signifikante Verbesserung des 1-Jahresüberlebens durch die präemptive TIPS-Implantation (Überleben 86 % vs. 61 %, p < 0,001) mit einer niedrigen „number needed to treat“ von 4 41 . Retrospektive Studien legen nahe, dass das Zeitfenster für den präemptiven TIPS unter Umständen auch deutlich über 72 Stunden hinaus erweitert werden kann 46 . Vor allem Patienten mit fortgeschrittener Lebererkrankung profitieren von einem präemptiven TIPS 42 , dies wird zusätzlich unterstützt durch eine multizentrische Beobachtungsstudie, die zeigt, dass ein präemptiver TIPS auch bei Patienten mit ACLF und akuter Varizenblutung das Überleben signifikant verbesserte 47 . In der letzten Baveno-Konsensus-Konferenz wurde zusätzlich zu den klinischen Indikationen auch ein Lebervenenverschlussdruckgradient (hepatic venous-portal gradient: HVPG) von über 20 mmHg als Indikation für einen präemptiven TIPS aufgenommen 48 .

#### TIPS in der Sekundärprophylaxe

Randomisierte Studien zur Sekundärprophylaxe der Varizenblutung zeigen, dass der TIPS sehr effektiv das Risiko einer Rezidivblutung senkt, ohne dass sich dies jedoch in einen Überlebensvorteil übersetzt 18 49 50 . Kommt es jedoch zur Rezidivblutung, besteht die klare Empfehlung, die TIPS-Implantation zu evaluieren, um das Risiko weiterer Rezidivblutungen zu senken 51 . Die Wahrscheinlichkeit einer Re-Blutung ist innerhalb von 2 Wochen am höchsten und der TIPS sollte möglichst frühzeitig erfolgen 49 . Nach 6 Wochen sinkt die Wahrscheinlichkeit einer Rezidivblutung 52 .

#### Portal-hypertensive Gastropathie

Zum Stellenwert des TIPS zur Therapie der rezidivierenden Blutung aus portal-hypertensiver Gastropathie (PHG) existieren lediglich unkontrollierte Untersuchungen, die aber nahelegen, dass der TIPS das Ausmaß der PHG und den Transfusionsbedarf effektiv reduzieren kann 53 54 . Falls trotz konservativer Therapie (optimalen NSBB-Einsatz, endoskopischer Therapie und ggf. Eisensubstitution) ein chronischer Blutverlust durch die PHG besteht, kann deshalb eine TIPS-Implantation in Betracht gezogen werden. Hierbei sollte die PHG von einem GAVE (gastric antral vascular ectasia)-Syndrom unterschieden werden. Bei GAVE-Syndrom ist die TIPS-Anlage nicht effektiv und nicht indiziert, sondern es wird die weitere Behandlung mittels *Argon* -Plasma-Koagulation empfohlen.

#### Ektope Varizen

Portal-hypertensive Blutungen aus gastrointestinalen Varizen außerhalb von Ösophagus oder Magen sind mit 1–5 % aller Varizenblutungen selten, dann aber oft schwer zu therapieren und mit einer hohen Letalität von bis zu 40 % verbunden 55 . Die TIPS-Implantation scheint in der Therapie und Prophylaxe ektoper Varizenblutungen trotz effektiver Drucksenkung weniger effektiv zu sein als bei Ösophagusvarizenblutungen. So sind in Fallserien Re-Blutungsraten von bis zu 25–46 % beschrieben worden. Eine zusätzliche endovaskuläre Embolisation der ektopen Varizen im Rahmen der TIPS-Anlage ist mit einer Senkung der Rezidivblutungsrate assoziiert 56 57 58 59 . Daher kann je nach Lokalisation und Blutungsschwere bei Patienten mit ektoper gastrointestinaler Varizenblutung die TIPS-Implantation kombiniert mit Embolisation der Varizen als effektive Maßnahme zur Blutstillung erwogen werden. Insbesondere Varizen, die von peripheren Ästen gespeist werden, sollten zusätzlich embolisiert werden.

### TIPS in der Therapie der vaskulären Lebererkrankungen

#### Budd-Chiari-Syndrom (BCS)

Alle Patienten mit einem akuten BCS sollen eine sofortige therapeutische Antikoagulation erhalten 60 . Da BCS-Patienten eine deutlich erhöhte Rate Heparin-induzierter Thrombopenien aufweisen, ist eine Therapie mit niedermolekularem Heparin einer Behandlung mit unfraktioniertem Heparin vorzuziehen, weil unter der Therapie mit unfraktioniertem Heparin generell ein erhöhtes HIT-Risiko vorliegt im Vergleich zu niedermolekularem Heparin 61 . In aktuellen Leitlinien wird ein stufenweises therapeutisches Vorgehen empfohlen, welches nach der Antikoagulation die Angioplastie der Lebervenen, die TIPS-Implantation und schließlich die Lebertransplantation vorsieht 52 61 . Der nächste Therapieschritt sollte bei entsprechendem vorherigen Therapieversagen – definiert als persistierender Aszites, Nierenversagen oder persistierend erhöhten Transaminasen – erfolgen 62 .

Der optimale Zeitpunkt einer TIPS-Anlage ist nicht definiert und hängt entscheidend vom klinischen Verlauf ab 63 64 . Ein erhöhter BCS-TIPS-Prognose Index Score (> 7) ist mit einer schlechteren Prognose vergesellschaftet und kann zur Indikationsstellung beitragen 65 . Bei fulminanten Verläufen ist die Mortalität deutlich erhöht, weshalb eine Notfall-TIPS-Anlage indiziert ist und diese Patienten in einem Lebertransplantationszentrum behandelt werden sollten 66 67 . Je schwerwiegender der klinische Verlauf, desto früher sollte die TIPS-Anlage erfolgen 66 . Zu beachten ist, dass nur 18 % der Patienten mit einem akuten BCS auf eine Antikoagulation ansprechen 68 . Entsprechend zeigt eine prospektive europäische Studie, dass bei 40 % der Patienten eine TIPS-Implantation notwendig wurde 69 . Nach TIPS-Anlage besteht eine gute Prognose mit 5-Jahresüberleben von 70–90 % ohne Lebertransplantation 65 66 67 69 70 71 72 . Als technische Besonderheit sollte der TIPS bei BCS maximal dilatiert werden, da dieser die Funktion der Lebervenen übernimmt und somit der Kongestion entgegenwirkt. Eine lebenslange Antikoagulation ist nach TIPS-Implantation oft erforderlich, da die überwiegende Mehrzahl aller Patienten mit BCS eine thrombophilen Diathese (am häufigsten MPN) aufweisen. Zudem ist je nach Grunderkrankung eine längerfristige Antikoagulation notwendig, mindestens 6 Monate bei nicht-länger bestehender Ursache (z. B. akute Pankreatitis, abdominelles Trauma) 51 .

Bei Patienten mit chronischem BCS und zirrhotischem Umbau ohne akute Kongestion, sollte die TIPS-Indikation entsprechend des Ausmaßes der Komplikationen der portalen Hypertension gestellt werden, wie bei Patienten mit Leberzirrhose ohne BCS 73 74 75 .

#### Pfortaderthrombose (PVT)

Zahlreiche Studien zeigen, dass die TIPS-Anlage auch bei chronischer PVT technisch möglich ist 76 77 78 79 80 81 82 83 . Bei der akuten nicht-zirrhotischen und nicht-malignen PVT sollte auch eine interventionelle Therapie mit transjugulärer (in TIPS-Technik) Lyse und/oder Thrombektomie und anschließender TIPS-Anlage erfolgen, wenn: a. unter der initial durchgeführten Antikoagulation (2 Wochen) keinen Rekanalisation erreicht werden konnte; b. in ausgewählten Fällen mit ausgedehnter kompletter Thrombose und drohender Mesenterialischämie 84 . Eine akute Thrombose hat bessere Chancen auf eine Rekanalisation, während das Vorliegen eines MPN die Wahrscheinlichkeit eines Therapieansprechens auf die Antikoagulation senkt 84 85 . Die sonografische Sichtbarkeit eines intrahepatischen Pfortaderastes erhöht den Punktionserfolg 81 . Nach Beendigung einer transjugulären Lysetherapie führt die TIPS-Implantation zur Steigerung der Flussrate und verhindert eine Rethrombosierung. Bei Nachweis einer thrombophilen Diathese (z. B. Faktor-V-Leiden, MPN) ist nach Rekanalisation der Pfortader eine lebenslange Antikoagulation erforderlich; ohne Nachweis einer Gerinnungsstörung (z. B. traumatische PVT) kann die Antikoagulation nach 6 Monaten beendet werden 52 .

Für die zirrhotische PVT werden hohe Raten (40–50 %) an spontaner Remission berichtet 86 87 . Trotzdem ist eine therapeutische Antikoagulation sinnvoll und indiziert 88 89 90 . Bei Progress der Thrombose unter einer etablierten Antikoagulation ist eine TIPS-Anlage in Erwägung zu ziehen 87 89 . Insbesondere vor einer geplanten Lebertransplantation oder bei einer höhergradigen Okklusion der Pfortader scheint ein solches Vorgehen sinnvoll. 80 91 92 . Für die übrigen Fälle gelten grundsätzlich die üblichen Indikationen zum TIPS 93 .

Patienten mit chronischer PVT und kavernöser Transformation, die portal-hypertensive Komplikationen aufweisen (z. B. Aszites, Varizenblutungen, Cholangiopathien), sollten in einem spezialisierten Zentrum zur TIPS-Anlage vorgestellt werden. Trotz der hohen technischen Herausforderung und eines erhöhten Interventionsrisikos kann beim Bestehen eines suffizienten portalen Gefäßzugangs, der sog. „landing zone“ (perfundiertes Gefäßlumen stromaufwärts der Thrombose), der TIPS in den meisten Fällen erfolgreich durchgeführt werden 77 81 83 94 95 . Hierbei sind vor allem die translienalen, transmesenterialen oder transhepatischen Zugangswege möglich, jedoch sollten diese technisch herausfordernden Eingriffe in spezialisierten Zentren erfolgen.

#### Sinusoidales Obstruktionssyndrom (SOS)

Die Studien zum Einsatz des TIPS beim SOS im Rahmen einer Stammzelltransplantation datieren aus den frühen 2000er Jahren und basieren auf dem Einsatz von unbeschichteten Stents. Die kleinen Fallserien von 3–10 Patienten zeigten zwar eine Reduktion der portalen Hypertonie aber keine Verbesserung der Prognose dieser Patienten 96 97 98 . Obwohl das SOS bei bis zu 2 % der Patienten nach einer Lebertransplantation aufzutreten scheint, fehlen relevante Studien zum Einsatz des TIPS bei SOS und solider Organtransplantation. Zusätzlich hat der Einsatz von Defibrotide in den letzten Jahren eine Mortalitätssenkung erbracht 99 . Trotzdem kann in Einzelfällen aber offenbar mit der TIPS-Anlage das Ausmaß der portal-hypertensiven Komplikation reduziert werden und können auch günstige Effekte in der Leberhistologie erreicht werden (Besserung der Fibrose, und/oder der sinusoidalen Stauung) 100 101 102 .

#### Porto-Sinusoidale Vaskuläre Erkrankung (PSVD)

Die Porto-Sinusoidale Vaskuläre Erkrankung (PSVD) vereint verschiedene histologische Veränderungen der Leber (obliterative portale Venopathie, nodulär regenerative Hyperplasie, inkomplette septale Fibrose) mit Zeichen einer portalen Hypertension 103 104 . Insgesamt liegen nur wenige Studien zur Behandlung der PSVD-induzierten portalen Hypertonie mittels TIPS-Anlage vor 105 106 107 108 109 110 . Die Indikation zum TIPS wurde dabei analog zu Patienten mit Leberzirrhose gestellt. In der größten Kohorte von 76 Patienten führte dieses Vorgehen zu einer Drittelung der Mortalität 110 . Aufgrund der besseren Leberfunktion sind hepatische Enzephalopathien und Leberversagen seltener, und ist die Prognose insgesamt besser.

#### Zugrunde liegende ätiologische Abklärung

Bei allen Patienten mit vaskulären Lebererkrankungen wird eine hämatoonkologische und gerinnungsphysiologische Abklärung empfohlen 52 .

### TIPS vor Operation und Lebertransplantation

#### Prophylaktische, präoperative TIPS-Implantation

Zum Einsatz des TIPS in prophylaktischer, präoperativer Intention vor viszeral-chirurgischen Operationen liegen aktuell nur retrospektive Daten sowie eine Metaanalyse vor 111 112 113 . Die bisherigen Studien sind bzgl. Patientenkohorten und Endpunkten sehr heterogen, bei denen nicht zwischen der TIPS-Anlage bei Komplikationen der Portalen Hypertension und der Prävention von Komplikationen nach Operation unterschieden wurde. Auch in der größten bislang publizierten Kohorte mit 66 präoperativen TIPS-Implantationen, die zu 68 Patienten ohne TIPS mittels Propensity Score Matching verglichen wurden, zeigte sich kein Vorteil in den Endpunkten postoperativer Komplikationen und Überleben 111 114 .

#### TIPS-Implantation vor Lebertransplantation

Die Indikationen zur TIPS-Anlage entsprechen den Indikationen bei Patienten, die nicht zur Lebertransplantation vorgesehen sind 115 116 117 . Die Listung zu einer Transplantation selbst ist keine alleinige Indikation für eine TIPS-Implantation, allerdings stellt die Listung für eine Transplantation auch keine Kontraindikation für die TIPS dar 116 117 . Insbesondere Patienten mit einer Pfortaderthrombose vor einer Lebertransplantation profitieren von einer TIPS-Anlage 92 118 119 .

Vor einer TIPS-Anlage sollten spezifische technisch-anatomische Gesichtspunkte geprüft werden. Hierbei sollten anatomisch die Lage der Lebervenen und die Cavaanatomie in Bezug zur Pfortader geklärt werden (Offenheit, Winkel, Trakt-Distanzen). Anatomische Varianten und Besonderheiten, insbesondere ein Z. n. LTX (Art der LTX, Größe des Transplantats, hypertrophiebedingte Lageveränderungen, Art und Position der cavalen und portalen Anastomosen, Offenheit der Lebervenen und Pfortaderäste) sollten im Vorfeld geklärt werden. Hierfür empfiehlt sich die Durchführung einer präinterventionellen Kontrastmittel (KM)-unterstützen Mehrphasen-CT der Leber. Es ist darauf zu achten, die Operabilität des Patienten durch eine entsprechende Positionierung des Stents nicht zu beeinträchtigen. Die Daten einer großen Transplantationsstudie zeigen, dass bei 13 % der mit einem TIPS behandelten Patienten der Stent zu weit in die Cava oder den rechten Vorhof oder bis in die extrahepatische Pfortader oder die Vena mesenterica superior platziert wurden. Hierdurch wurde die Durchführung der Lebertransplantation technisch schwieriger, was einen signifikanten Einfluss auf das 1-Jahresüberleben (71 % vs. 92 %, p = 0.01) hatte. Diese Anforderungen sind nicht nur auf Transplantationspatienten zu beschränken, sondern sollten bei allen Patienten angestrebt werden, da eine optimale Position des Stents für eine gute und anhaltende Shuntfunktion von Bedeutung ist und auch keine zusätzliche technische Herausforderung darstellt. Eine UNOS-Datenbankanalyse aus dem Jahr 2017 hat die Ergebnisse von mehr als 32 000 Lebertransplantationen (davon 1300 Patienten mit einer zuvor durchgeführten TIPS-Anlage) ausgewertet 117 . Diese Studie zeigte zusätzlich, dass die Anlage eines TIPS die Wartezeit bis zur Lebertransplantation verdoppelte (183 vs. 408 Tage, p < 0.001) 117 120 . Der mögliche Effekt einer TIPS-Anlage auf die Organallokation sollte daher im Einzelfall bedacht werden.

## Patientenselektion und Evaluation

### Rolle des Body-Mass-Index

Prospektive Studien, die eine klare Festlegung eines sehr hohen oder sehr niedrigen BMI als Ausschlusskriterium für eine TIPS-Anlage rechtfertigen würden, fehlen. Das Vorliegen einer Sarkopenie (mittels Schnittbildgebung erhoben) stellt einen unabhängigen Risikofaktor für die Entwicklung einer HE nach TIPS-Anlage dar 121 122 . Auch konnte gezeigt werden, dass eine Sarkopenie die Überlebenswahrscheinlichkeit nach TIPS beeinflusst 123 . Grenzwerte für den BMI sind nach heutigem Wissenstand nicht zu formulieren. Man kann annehmen, dass analog zu Lebertransplantation, Patienten mit einem BMI < 18,5 oder > 45 kg/m ^2^ mit einem erhöhten Risiko im Rahmen von Interventionen rechnen müssen 124 .

### Rolle des Alters

Ein hohes Patientenalter ist ein negativer Prädiktor für den postinterventionellen Verlauf 125 . Insbesondere nimmt das HE-Risiko mit dem Alter zu. Bei Patienten ≥ 65 Jahren ist der post-interventionelle Verlauf komplikativer 126 . Dies muss bei der Indikationsstellung berücksichtigt werden. Allerdings spielt das Alter keine Rolle auf die Krankenhaussterblichkeit nach TIPS, sogar in den Gruppen älter als 80 Jahren 31 , deshalb ist das kalendarische Alter kein hartes Kriterium, um einen TIPS nicht einzusetzen.

### Kardiale Diagnostik vor der TIPS-Anlage

Die kardiale Diagnostik vor TIPS-Anlage sollte die Frage klären, ob eine schwere links- als auch rechtsventrikuläre systolische oder diastolische Dysfunktion, oder eine signifikante pulmonale Hypertonie vorliegt. Mindestanforderungen an den echokardiografischen Befund sind die Darstellung der Dimensionen der Herzhöhlen, globale sowie regionale Pumpfunktion des linken und rechten Ventrikels, Wanddicke des linken Ventrikels, Morphologie und Funktion der Herzklappen, Abschätzung des systolischen pulmonalarteriellen Spitzendrucks (invasive Abklärung bei PAPsys> 45 mmHg), Rechtsherzbelastung und rechtsventrikuläre Herzinsuffizienz, Veränderungen des Perikards sowie größere strukturelle Veränderungen bei kongenitalen Fehlbildungen des Herzens. Es wird für die TIPS-Anlage eine untere Grenze der Ejektionsfraktion von 40 % empfohlen. Diese Grenze ist willkürlich, da keine entsprechenden Studienergebnisse vorliegen. Die nicht-invasive (echokardiografische) Bestimmung des pulmonalarteriellen Druckes ist nicht sehr zuverlässig. Deshalb sollte bei entsprechendem klinischem Verdacht eine Druckmessung mittels Rechtsherzkatheter durchgeführt werden, wobei bei einer Pulmonalarterien Hypertonie eine TIPS-Implantation relativ kontraindiziert ist ( Tab. 1 ).

**Tab. 1:** Kardiale Diagnostik vor TIPS-Implantation.

Messgröße	Normwerte
**2D- oder 3D-Volumina**	Enddiastolisches Volumen: 35–75 ml/m ^2^
	Endsystolisches Volumen: 12–30 ml/m ^2^
**LV-Diameter (M-Mode, 2 D)**	Enddiastolischer Diameter: 22–32 mm/m ^2^
	Endsystolischer Diameter: 14–21 mm/m ^2^
**Septum- und Hinterwanddicke**	interventrikuläres Septum: 6–10 mm
	linksventrikuläre Hinterwand: 6–10 mm
**Linksventrikuläre Auswurffraktion**	> 55 % (minimum > 40 %)
**Regionale Wandbewegungsstörung**	nein
**Linksatriales Volumen**	< 34 ml/m ^2^
**E/A**	< 1,5
**E/e'**	< 1,0
**mittlere PA-Druck**	< 30 mmHg
**PA-Druck (sPAP)**	< 55 mmHg

Die Bestimmung des natriuretischen Peptids vom Serum-N-terminalen Pro-B-Typ (NT-proBNP) ist als Screening-Parameter für das Vorliegen einer Herzinsuffizienz sinnvoll 127 . Anhand der NT-proBNP-Konzentration konnte in einer prospektiven Studie mit 100 Patienten das Risiko einer kardialen Dekompensation nach TIPS vorhergesagt werden, jedoch nicht die Sterblichkeit; die Autoren schlugen einen Wert von unter 125 pg/ml als sicher für die TIPS-Implantation vor 128 . In diesem sogenanntem Toulouse-Algorithmus sollte bei höheren NT-proBNP-Konzentrationen eine detaillierte Analyse der diastolischen linksventrikulären Funktionsparameter durchgeführt werden, um bei E/A < 1.5; E/e' < 10; LAVI < 34 ml/m ^2^ ) ein niedriges Risiko und bei E/A > 1.5 oder E/e' > 10 oder linksatrialer Volumenindex (LAVI) > 34 ml/m ^2^ ein hohes Risiko einer kardialen Dekompensation nach TIPS zu attestieren. In derselben Studie zeigten insbesondere auch Patienten mit höhergradiger Aortenklappenstenose hohe Raten an kardialer Dekompensation nach TIPS-Implantation 128 .

### Bildgebung vor TIPS-Anlage

Obwohl die Duplex-Ultraschalluntersuchung in der Regel viele der für die TIPS-Implantation erforderlichen Informationen liefern kann, ist zur Eingriffsplanung und zum Ausschluss von hepatischen Raumforderungen, einer Pfortaderthrombose, oder relevanten Stenose der A. hepatica, sowie zur Beurteilung des Ausmaßes der portosystemischen Kollateralen eine kontrastmittel-gestützte multi-phasische CT- oder MRT-Untersuchung notwendig.

### Grenzwerte für die Gerinnung und Substitution mit Gerinnungsfaktoren

Bei 40 % der Patienten mit Leberzirrhose besteht eine Thrombopenie (< 80 000/mm ^3^ ). Das Ausmaß der Thrombopenie korreliert mit dem Child-Pugh-Stadium. Trotzdem soll bei Leberzirrhose keine Gerinnungssubstitution routinemäßig erfolgen. Eine Transfusion sollte bei TZ ≤ 50 000/mm ^3^ und INR ≥ 2,5 erwogen werden, obwohl es wenig direkte Evidenz hierfür gibt 129 130 .

### Kontraindikationen

Bei Patienten mit refraktärem Aszites ist die HE multifaktorieller Genese (eingeschränkte Leber- und Nierenfunktion, Exsikkose 8 , Infekt, Hypoglykämie, spontane portosystemische Shunts). Auch die Sarkopenie scheint einen deutlichen Einfluss auf die Entwicklung der HE nach TIPS zu haben 121 . Daher unterscheidet sich das kumulative Auftreten der ersten HE-Episode nicht zwischen den Behandlungsgruppen (TIPS vs. wiederholte Parazentesen) 8 . Allerdings erhöht der TIPS die Anzahl der HE-Episoden insgesamt sowie die Anzahl schwerer HE-Episoden 8 . Auch eine kürzlich publizierte randomisiert-kontrollierte Studie zeigte keinen Unterschied im Auftreten der HE zwischen Patienten, die einen TIPS erhielten, verglichen mit denen, die nur durch Parazentesen und Albumininfusion behandelt wurden (34 % HE im ersten Jahr in beiden Gruppen) 2 . Es ist offensichtlich, dass eine aktive und insbesondere unkontrollierte Infektion oder Sepsis eine Kontraindikation für die TIPS-Implantation ist, und das Fenster für die TIPS-Anlage soll nach Möglichkeit nach Ausheilung oder zumindenst Kontrolle der Infektion gewählt werden.

Bei Vorliegen von Risikofaktoren für eine HE (höheres Lebensalter > 70 Jahren, vorherige HE-Episoden, mittlerer arterieller Blutdruck < 80 mmHg, MELD-Score > 15), aber dringlicher TIPS-Indikation ist eine limitierte Drucksenkung des portosystemischen Druckgradienten (z. B. 30–50 %) anzustreben. Eine prospektive Studie konnte zeigen, dass ein kleinerer Stentdiameter (8 mm) im Vergleich zu größeren Stentdiametern (10 mm) die HE-Rate nach TIPS-Anlage deutlich senkt 131 . Die Daten dieser chinesischen Studie stimmen mit der Rate an HE in der deutschen Blutungsstudie mit 8 mm beschichteten TIPS von ca. 18 % überein 49 , wobei die Verwendung von 10 mm Stents ca. doppelt so häufig HE-Episoden zeigte, und mit der niederländischen Studie mit 10 mm beschichteten TIPS übereinstimmt 50 . Auch wenn diese Studien an Patienten mit Varizenblutungen vorgenommen wurden, zeigen sie dennoch, dass kleinere Stentdurchmesser zu bevorzugen sind.

Ein Bilirubin über 3 mg/dl korreliert mit einer erhöhten Letalität nach TIPS-Anlage 132 . Jede Erhöhung des Bilirubins um 1 mg/dl über 3 mg/dl hinaus steigert das Risiko der 30-Tages-Letalität um 40 % 133 . Deshalb wird eine Erhöhung des Gesamtbilirubins über 3 mg/dl allgemein als relative und eine Erhöhung über 5 mg/dl als absolute Kontraindikation einer TIPS-Anlage zur Behandlung von Aszites angesehen 134 . Zudem ist die Kombination aus der Thrombozytenzahl (< 75Tsd/l) und Bilirubin (> 3 mg/dl) bei Patienten mit refraktärem Aszites als prognostisch ungünstiges Zeichen anzusehen 135 , obwohl auch schlechtere Patienten profitieren 8 . Diese ungünstige Konstellation stellt zwar keine absolute Kontraindikation dar, muss aber im individuellen Therapiekonzept gewürdigt werden. In der DGVS-Leitlinie des Jahres 2017 ist TIPS-Implantation zur Aszitestherapie bei vorbestehender chronischer hepatischer Enzephalopathie ≥ Grad 2 (ohne Auslöser) oder einem Serum-Bilirubin > 5 mg/dl 136 in der Regel kontraindiziert.

Basierend auf retrospektiven Beobachtungsstudien haben diese Kriterien im Kontext eines Notfall-TIPS bei akuter Varizenblutung keine absolute Gültigkeit 137 . Für die Implantation eines präemptiven TIPS nach initialer Varizenblutung stehen mehr Daten zur Verfügung. In der ersten Studie, die die Implantation eines so genannten early-(präemptiven)-TIPS untersucht hat, wurden Patienten (n = 63) mit Child-Pugh B und aktiver Blutung oder Child-Pugh C 10 bis 13 Punkte eingeschlossen. Übliche Kontraindikationen, wie hepatische Enzephalopathie oder Ikterus, wurden in dieser Studie nicht als Ausschluss-Kriterien gewertet. In der Tat wurden Patienten mit eindeutigem Ikterus (Bilirubin 4,4 +/– 4,9 mg/dL in der Kontrollgruppe und 3,7 +/–4,8 mg/dL in der TIPS-Gruppe) und mit vorheriger hepatischer Enzephalopathie (keiner in der Kontrollgruppe und 6 Patienten in der TIPS-Gruppe) eingeschlossen, trotzdem hatten Patienten mit TIPS-Implantation einen Überlebensvorteil. Weitere Studien mit vergleichbaren Einschlusskriterien ergaben ähnliche Ergebnisse 19 138 . In der jüngsten Meta-Analyse wurden diese Vorteile des TIPS bei Hoch-Risiko-Patienten bestätigt 139 .

Zum Stellenwert des TIPS im onkologisch-hepatologischen Therapiekonzept für Patienten mit dekompensierter Leberzirrhose und HCC existieren keine randomisierten kontrollierten Studien. In einer Fall-Kontrollstudie war die Anlage eines TIPS bei Patienten mit HCC mit einer geringeren Rate Tumor-gerichteter Therapien assoziiert im Vergleich zur Gruppe ohne TIPS. Patienten mit HCC und TIPS erhielten signifikant seltener eine transarterielle Chemoembolisation (TACE). Dies übersetzte sich in ein signifikant schlechteres Überleben der Patienten mit HCC und TIPS 140 , sodass womöglich eine onkologische Untertherapie vorlag. Eine Fallserie von 40 Patienten mit HCC und TIPS schloss 9 Patienten mit ein, die auch eine TACE erhielten. Diese Patienten hatten keine erhöhten ischämischen Komplikationen nach TACE 141 . Auch andere Fallserien sprechen dafür, dass die TACE und andere interventionelle Tumortherapien bei vorliegendem TIPS durchaus effektiv und sicher sind 142 , wenn auch womöglich mit einem etwas erhöhten Komplikationsrisiko assoziiert ist 143 . Gerade bei Patienten mit potenzieller kurativer Therapieoption durch eine Transplantation muss beachtet werden, dass eine Tumorstreuung durch den TIPS theoretisch vorstellbar ist. Auch hierzu liegen keine größeren Datensätze vor. In der oben genannten Fall-Kontroll-Studie entwickelte lediglich einer von 40 Patienten Lungenmetastasen und dies erst 7 Jahre nach der TIPS-Anlage 141 . Eine andere Gruppe beobachtete Fernmetastasen in 7 von 58 Patienten (12 %) mit HCC und TIPS 148 .

An manchen Zentren wird im Rahmen individueller Therapiekonzepte ein TIPS bei gleichzeitigem HCC vor allem bei Patienten in Betracht gezogen, bei denen die portal-hypertensive Dekompensation das klinisch führende Problem darstellt und eine Lebertransplantation nicht in Frage kommt. Nach Rekompensation durch den TIPS kann gelegentlich auch eine effektivere HCC-Therapie angeschlossen werden.

### Technische Durchführung der TIPS-Anlage

### Notwendige Ausstattung für die Implantation

Gerätetechnische Voraussetzungen sind ein hochauflösender Flachdetektor und Röntgen-Anlagen mit der Möglichkeit zur digitalen Subtraktionsangiografie. Ein US-Gerät mit einem linearen 7,5–12 MHz wird zur US-geführten jugulären Venenpunktion sowie ein sektorieller 3,5–5 MHz-Schallkopf zur US-Führung der TIPS-Punktionsnadel werden zur Portalvenenpunktion standardmäßig verwendet 81 145 . Weiterhin muss eine Möglichkeit der Sauerstoffversorgung gegeben sein. Ein Überwachungsmonitor mit der kontinuierlichen Möglichkeit der Messung von EKG, Herzfrequenz, Blutdruck (nichtinvasiv), Sauerstoffsättigung und Möglichkeit der invasiven Druckmessung in Gefäßsystemen muss vorhanden sein 146 .

Prozedurbedingtes Equipment sind eine TIPS-Punktionsnadel, wobei zwischen offenlumigen Nadeln (Colapinto) und Stilettnadelsystemen (Rösch-Uchida) unterschieden wird. Eine vergleichende Studie zu den unterschiedlichen Nadelsystemen konnte keinen Unterschied zwischen den beiden zeigen 147 . Zur Sondierung der Lebervenen und der Pfortader werden unterschiedliche angiografische Katheter und Drähte (hydrophile und versteifte Drähte) benötigt. Weiterhin sollten 8–10 mm Stentgrafts (ePTFE-gecoverte Stents) in unterschiedlichen Längen (4–8 + 2 cm) und unterschiedliche Angioplastieballons (4 bis ≥ 10 mm Durchmesser) vorhanden sein 148 149 150 151 152 153 . Für den möglichen Verschluss von portosystemischen Kollateralgefäßen werden Coils, Plugs bzw. Flüssigembolisate (z. B. Cyanoacrylat) verwendet 154 155 .

### Strukturelle Voraussetzungen der Klinik

Voraussetzungen zur Implantation eines TIPS bestehen in der Vorhaltung einer Intensivstation mit entsprechender Expertise bei Behandlung von Patienten mit einer dekompensierten Leberzirrhose. Weiterhin wird eine viszeralchirurgische Abteilung mit Expertise in komplexer Leberchirurgie und/oder Lebertransplantation als Voraussetzung für eine TIPS-Implantation empfohlen. Beide Einrichtungen sind insbesondere im Kontext des Komplikationsmanagements wichtig. Die Rate an relevanten Komplikationen oder Todesfällen wird insgesamt als gering (< 5 %) eingeschätzt 156 157 . Die Studie von Sawar et al. 158 und die DRG-Daten aus Deutschland geben demgegenüber ein deutlich höhere Gesamt- und Krankenhausletalität bei Patienten nach TIPS-Implantation an. Die Komplikationsrate scheint nach dieser Arbeit wesentlich mit der Mindestanzahl der TIPS-Implantationen pro Jahr zusammenzuhängen 31 158 . Die adjustierte Letalitätsrate war bei Zentren mit einer Implantationsrate zwischen 1–19 TIPS/Jahr signifikant höher (10 % vs. 7 %) verglichen mit den Zentren, die ≥ 20 TIPS/Jahr implantierten 158 . Auch in Deutschland ist die jährliche Zahl der TIPS-Anlagen mit der Krankenhausmortalität eng assoziiert und ≥ 20 TIPS/Jahr mit einem besseren Überleben assoziiert 31 . Eine deutschlandweite Befragung von 76 Zentren mit TIPS-Implantation ergab, dass die Anzahl der Implantationen zwischen 5 und 90 pro Jahr schwankten, im Median bei etwa 28 TIPS-Implantationen pro Jahr liegt, sodass bei der Mehrzahl von einer ausreichend hohen Rate ausgegangen werden kann 159 . Aufgrund der technischen Herausforderungen sollte eine TIPS-Anlage beim PVT und BCS nur an spezialisierten Zentren mit hoher TIPS-Frequenz durchgeführt werden 160 161 162 .

Empfohlen zur Senkung der Komplikationsrate und Steigerung der Erfolgsrate ist die fachübergreifende interventionell-radiologisch- und internistisch-interventionelle Expertise (TIPS-Board o. ä.). Auch zur Senkung der prozedurbedingten Strahlendosis kann der Einsatz des Ultraschalls beitragen. Derzeit liegen nur eingeschränkte Daten bzgl. der Strahlenexposition des Patienten und des Operateurs während der Anlage eines TIPS vor. Ebenso gibt es vom BfS derzeit keine Referenzwerte für diesen Eingriff. Miraglia et al. konnten allerdings an einem kleinen Kollektiv zeigen, dass das mittlere Dosisflächenprodukt 235 ± 198 Gy cm ^2^ beträgt 163 . Dabei lag die mittlere effektive Dosis für den Operateur bei 1,40 ± 2,68 μSv 163 . Darüber hinaus konnte das Team ebenso wie David et al. eine signifikante Reduktion der Strahlenexposition durch die Ultraschall-gesteuerte Punktion des Pfortadersystems im Gegensatz zu einem fluoroskopisch gesteuerten Zugang nachweisen 164 165 .

### Darstellung der Gefäße bei der Anlage

Bei regulärer Anatomie ist der Standardzugang die rechte Lebervene. Die Pfortader/der rechte Pfortaderhauptstamm liegt kaudoventral der rechten Lebervene und ist damit über die von kraniodorsal kommende Punktionsnadel i. d. R. gut erreichbar. Bei Okklusion der rechten Lebervene, oder bei abnormer Lage des Organs und des Pfortadersystems oder bei Teilokklusion des Pfortadersystems kann ein Zugang über die mittlere, die linke Lebervene oder gar direkt über die Vena cava inferior (DIPS, Direkter TIPS) nötig sein. Ist keiner dieser Zugangswege möglich, kann die Möglichkeit eines Hybrideingriffs mittels perkutaner PV-Lebervenen/VCI-Punktion mit transjugulärem Rendezvous erwogen werden 166 .

**Abb. 3:**
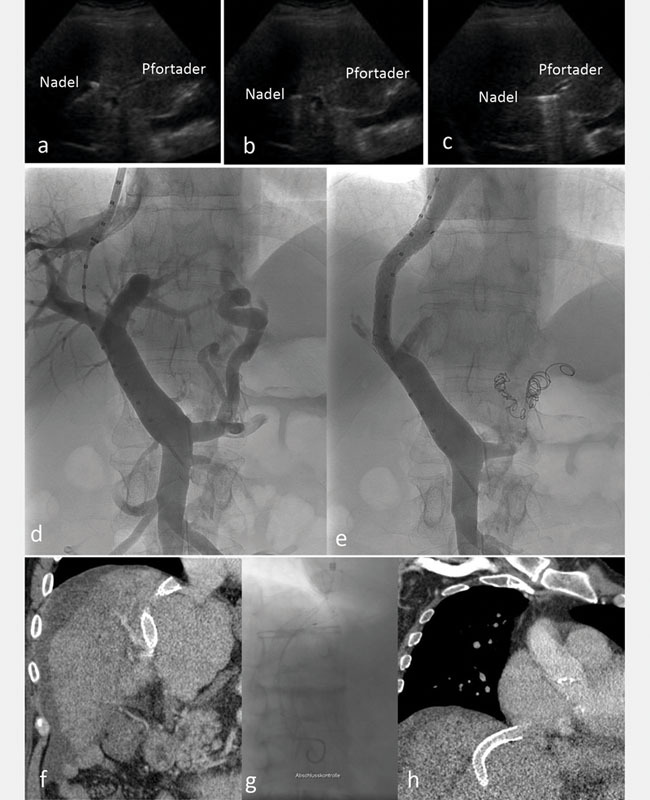
Abbildungen **a–c** zeigen die Ultraschallgesteuerte Punktion der Pfortader. Die Nadel wird verfolgt von Ausgang aus der Lebervene ( **a** ), über den Parenchymtrakt ( **b** ) und bis in die Pfortader ( **c** ). Die Angiographiebilder **d** und **e** zeigen eine Standardmessung der Länge ( **d** ) und Implantation des Stents inklusive einer Embolisation von der die Varizenspeisende V. Coronaria Ventriculi ( **e** ). In den Bildern **f, g** und **h** sieht man einen dysfunktionalen TIPS. Die CT zeigt, dass der eingebrachte TIPS der kranialen Lebervene unmittelbar aufsteht ( **f** ). Durch Verlängerung nach zentral mittels eines bare metal-Stents bis an den Übergang der Lebervene zur VCI ( **g** ) konnte der Ausflusstrakt optimiert werden ( **h** ). [rerif]

Im Fokus stehen Techniken, die den Punktionsvorgang direkt sichtbar machen, hierzu gehören der intravaskuläre 153 und der abdominale Ultraschall 156 158 . Die Steuerung mittels abdominalen Ultraschalls ist einfach, überall verfügbar und auch i. d. R. bei adipösen Patienten oder bei Patienten mit Aszites (ggf. nach Parazentese) durchführbar. In der interkostalen schrägen Schnittebene ist die Nadel zu erkennen und in Richtung des rechten Pfortaderhauptstammes zu lenken ( Abb. 3a–c ). Mittels perkutanen Ultraschalls kann die Anatomie der Lebervenen und der Pfortader in Beziehung zur Position der TIPS-Punktionsnadel gesetzt und eine dreidimensionale Orientierung für die Stichrichtung auf die Pfortader erreicht werden 167 . Änderungen der Lagebeziehung zwischen Lebervene und Pfortader durch das starre Punktionsbesteck oder aber während des Punktionsvorgangs sind insbesondere bei Patienten mit Aszites oft erheblich und können durch die Echtzeitdarstellung erfasst werden. Dadurch können Nadelführung und Angulation sofort angepasst werden. Durch die US-Führung wird die Anzahl der Punktionsversuche im Vergleich zu anderen Orientierungsmaßnahmen reduziert, die Komplikationen minimiert und die Prozedurdauer inklusive der Durchleuchtungszeit verkürzt, bzw. auch die Strahlenexposition für das Interventionsteam und den Patienten reduziert 163 . Durch Ultraschallsteuerung konnten die Anzahl der Kapselperforationen von 34 % auf 9 % reduziert werden 153 . Obwohl seit Jahrzehnten der abdominale Ultraschall zur Steuerung eingesetzt wird, existieren bislang keine größeren Studien über dessen Effektivität. Die indirekte Portografie erbringt keine dreidimensionale Darstellung des Punktionsweges, zudem fehlt die Echtzeitdarstellung der Punktion. Die indirekte Portografie kann aber bei fehlender Darstellbarkeit im Ultraschall eine Alternativmethode zur Punktionsorientierung darstellen. Insbesondere bei Vorliegen eines Budd-Chiari-Syndroms oder einer thrombosierten Pfortader ist die Ultraschall-gesteuerte Führung extrem hilfreich ( Abb. 3a–c ).

### Punktion der Pfortader

Die Punktion der Pfortader ist der technisch anspruchsvollste Teil der Intervention, der über Erfolg und Komplikationen entscheidet und sollte unter US-Führung erfolgen. Die Steuerung dieses Vorgangs kann zusätzlich durch Bildfusion und dreidimensionale Rekonstruktion der Gefäßanatomie von unterschiedlichen Bildgebungsverfahren unterstützt werden.

Ziel der Punktion sollte der rechte Pfortaderhauptstamm sein, da er in der Regel sehr gut sonografisch einstellbar, von der rechten Lebervene gut erreichbar und zu allen Seiten gedeckt intrahepatisch punktierbar ist. Zwei post-mortem Studien zeigten, dass die Pfortaderbifurkation nur bei 53–60 % der Patienten intrahepatisch liegt 159 163 . Um Blutungskomplikationen zu vermeiden, sollte also eine Punktion der Pfortaderbifurkation vermieden werden. Als Zielpunkt dient vielmehr der rechte Hauptstamm in einem genügend großen Abstand von etwa 2 cm zur Bifurkation 163 164 . Der Abstand der Punktionsstelle von der Bifurkation kann am besten durch eine angulierte Angiografie (25 Grad RAO) beurteilt werden. Bei schlechter Zugänglichkeit oder Verschluss des rechtsseitigen Pfortaderhauptstammes kann der linksseitige Pfortaderhauptstamm ebenfalls 2 cm von der Bifurkation punktiert werden. Mehrere chinesische Arbeiten empfehlen generell die Punktion des linken Hauptstammes, da hierdurch eine geringere Inzidenz der hepatischen Enzephalopathie und bessere Offenheitsrate gesehen wurde 165 166 167 168 . Diese Empfehlung hat sich nicht durchsetzen können, da die vermeintlich verminderte HE-Rate nicht durch eine plausible, hämodynamisch fundierte Rationale erklärbar ist, die linksseitige TIPS-Anlage in der Regel schwierig ist, und man mit mehr Komplikationen (Kapselperforationen) rechnen muss.

Wenn die Standard-TIPS-Anlage von der rechten Jugularvene wegen steil abgehender Lebervenen oder eines weit lateral liegenden rechten Pfortaderhauptstammes nicht möglich ist, bietet sich ein Versuch von der linken V. jug. interna an. Dieser Zugangsweg gestattet die Katheterisierung steil abgehender Lebervenen und erleichtert die Punktion bei sehr weit ventral und lateral gelegener Pfortader. Der Eingriff von links ist schwieriger und deshalb nur als 2. Wahl nach gescheitertem Eingriff von rechts zu empfehlen.

### Prozedurale Komplikationen

Gravierende Komplikationen sind bei TIPS-Prozeduren selten und bestehen im Wesentlichen aus Fehlpunktionen in das Abdomen, der Gallenwege oder der Leberarterie sowie Infektionen 169 170 . Die Fehlpunktion durch die Leberkapsel kann durch eine Ultraschallsteuerung minimiert werden 153 . Falls dies dennoch erfolgt, kann bei Patienten mit hohem Blutungsrisiko eine Embolisation des Parenchymtraktes inklusive der Perforationsstelle der Leberkapsel durchgeführt werden. Punktionen der Gallenblase, Gallenwege oder Arterienäste sind nicht immer vermeidbar. In einer post-mortem-Studie wurde gezeigt, dass bei 36 % der Punktionen Gallengänge oder Äste der Leberarterie getroffen und verletzt wurden 159 . Aus diesen Gründen sollte vor der Stent-Implantation bei V. a. Fehlpunktion von Gallengänge oder Leberarterienäste eine Kontrastmitteldarstellung des Parenchymtraktes erwogen werden 171 . Falls sich eine entsprechende Kommunikation zeigt, muss dieser Zugang aufgegeben und eine neue Punktion der Pfortader durchgeführt werden.

Unerkannte Fehlpunktionen mit ggf. Fistelbildungen in die Gallengänge können zur Hämobilie und Bilhämie führen. Diese Komplikation ist meistens selbstlimitierend. Die Verletzung der Leberarterie kann einen fatalen Verlauf zur Folge haben. Bei vorliegendem Verdacht und entsprechender Symptomatik (schwere Hämobilie, Anstieg der Transaminasen, pulsierender Blutfluss im Portalsystem, Hämatombildung, Blutung in die Gallenblase) sollte die Indikation zur Arteriografie rasch gestellt werden. Bei arteriellem Blutungsnachweis sollte eine transarterielle Embolisation durchgeführt werden. Infektiöse Komplikationen in Form von Septikämie und Fieber treten in 3,9 % auf und nehmen mit zunehmender Dauer des Eingriffs zu 169 170 .

### Stenttypen für die TIPS-Anlage

PTFE-beschichtete Stentgrafts reduzieren nachweislich die Okklusionsrate im Vergleich zu unbeschichteten Stents („bare metal stents“; BMS). In einer prospektiven Studie konnte das Risiko der TIPS-Dysfunktion bei PTFE-beschichteten Stentgrafts gegenüber BMS mit einer Odds-ratio (OR) von 0,6 (95 %CI 0,38–0,96) signifikant gesenkt werden 152 . In einer retrospektiven Studie bei Patienten, die einen TIPS aufgrund therapierefraktären Aszites erhielten, konnte neben einer deutlich höheren 1-Jahres-TIPS-Offenheitsrate von 79,9 % in der PTFE-beschichteten Stentgraftgruppe gegenüber 23,1 % in der BMS-Stentgruppe auch ein 2-Jahres-Gesamtüberleben von 85,9 % gegenüber 69,5 % dokumentiert werden 172 . Eine weitere retrospektive Studie zeigte auch bei Blutung die Überlegenheit von PTFE-beschichteten Stentgrafts gegenüber den BMS-Stents 173 . In einer Meta-Analyse, die insgesamt 4 RCTs, 2 prospektive Studien und 8 retrospektive Studien umfasste, wurde der Vorteil der PTFE-beschichteten Stentgrafts bestätigt. Die 1-Jahres-Offenheitsrate für PTFE-beschichtete Stentgrafts wies eine OR von 4,75 (95 %CI 3,31–6,79; p < 0,001) auf. Auch für das 1-Jahres-Überleben konnte in der PTFE-beschichteten Stentgraftgruppe ein signifikanter Vorteil mit einer OR von 1,85 (95 %CI 1,44–2,38; p < 0,001) beobachtet werden 174 .

Der einzige für die TIPS zugelassene PTFE-beschichtete Stentgraft weist gegenüber anderen selbstexpandierbaren Standardstentgrafts den Vorteil auf, dass eine kontrollierte Expansion auf den gewünschten Diameter (8–10 mm) erfolgen kann und diese unverändert bleibt. Wenn notwendig, kann im Verlauf eine kontrollierte und schrittweise Expansion auf den Maximaldurchmesser erfolgen. Demgegenüber nehmen übrige selbstexpandierbare Standardstentgrafts im Verlauf über die Zeit immer ihren Nominaldiameter an, unabhängig von der initial per Ballondilatation gewählten Weite, sodass bei Wahl eines großen Stentdiameters möglicherweise der optimale Shuntdiameter im Verlauf überschritten wird, mit konsekutiven Auswirkungen auf den portosystemischen Gradienten und die HE-Rate 175 . Die klinische Relevanz der Eigenschaft der kontrollierten Expansion wird durch retrospektive Studienergebnisse angedeutet, die zeigen, dass das mediane Überleben nach Dilatation auf 8 mm gegenüber einer Dilatation auf 10 mm signifikant länger ist (34 versus 18 Monate). Die HR der 1-Jahres-Letalität betrug 2,0 (95 % CI 1,1–3,5) nach Dilatation auf 10 mm gegenüber einer Dilatation auf 8 mm 176 . Neuere Stents mit kontrollierter Expansion scheinen mindestens in einer ersten Studie klinisch einen Vorteil zu bringen 177 .

### Stentlänge

Zur Reduktion der Intimaproliferation in der drainierenden Lebervene, die früher eine häufige Ursache der Shuntinsuffizienz darstellte, wird seit Verfügbarkeit des PTFE-beschichteten Stents empfohlen, diesen am cranialen Ende bis zur Einmündung der Lebervene in die V. cava inferior zu platzieren 178 179 180 181 . Die exakte Bestimmung der notwendigen Stentlänge wird am besten durch eine simultane Kontrastmittelinjektion in die Pfortader bzw. Milzvene und in die V. cava-inferior gewährleistet ( Abb. 3d, e ). Auf diese Weise kann man die Start- und Landezone des Stents erkennen und die notwendige Stentlänge ausmessen ( Abb. 3d, e ). Die Markierungen des Messkatheters vereinfachen die Längenbestimmung und eliminieren Messfehler aufgrund des gebogenen Verlaufs des Shunttraktes. Falls die Länge des Shunts planimetrisch auf dem Angiografiebild gemessen wird, sollte aufgrund des zumeist gebogenen Verlaufs ein Zuschlag in der Stentlänge von 1cm erfolgen 182 .

### Druckmessung und deren Bedeutung

Da die Druckmessung eine erhebliche Relevanz auf das weitere Vorgehen haben kann, ist die Zuverlässigkeit der Messung von großer Bedeutung. Bei Unsicherheit der elektronischen Messung über einen Drucksensor (z. B. schlechte Reproduzierbarkeit) kann eine direkte hydrostatische Messung mittels „Wassersäule“ vorgenommen werden. Die Umrechnung in mmHg erfolgt durch Division (cm Wassersäule) mit dem Faktor 1,3. Die Druckmessung in der Pfortader soll mittels eines Mehrlochkatheters (oder Pigtailkatheter) erfolgen. Die Messung in der V. cava inferior erfolgt in der Regel über die liegende Schleuse, welche vor der Druckmessung gespült und auf Dichtigkeit geprüft werden muss. Vor der Spülung muss aspiriert werden, um einen Thrombus in der Schleuse auszuschließen. Falls die Aspiration blockiert ist, muss die Schleuse herausgezogen und gespült werden. Die Druckspülung des Systems vor der Messung sollte bei freier Durchgängigkeit zu einem prompten Anstieg bzw. Abfall des Druckes führen, andernfalls ist von einer Verlegung bzw. Wandadhärenz auszugehen. Die Druckkurven sollten einen horizontalen Verlauf über mindestens 1 Minute aufweisen.

Der Pfortaderdruck sollte im Pfortaderhauptstamm oder Confluens venae portae gemessen werden. Die Subtraktion des Referenzdruckes dient dem inneren Abgleich, der den äußeren Nullpunkt der Druckmessung relativiert und verschiedene Hydratationszustände des Patienten neutralisiert. Die Bestimmung des freien Lebervenendrucks ist aufgrund der Platzierung des TIPS nicht mehr möglich, weswegen der ebenfalls in Studien untersuchte und von verschiedenen Expertenforen empfohlene Druck der Vena cava inferior am Eintritt der Lebervenen gemessen werden soll 134 .

Die Messung des indirekten portosystemischen Druckgradienten (Leberveneverschlussdruckmessung – HVPG) vor TIPS-Anlage wird zur Überprüfung der präprozeduralen Druckverhältnisse empfohlen. Die Messung des Drucks in der Vena cava inferior, der freie Lebervenendruck und der geblockte (wedge-) Druck, als Surrogat für den Pfortaderdruck, sollten in diesem Zusammenhang bestimmt werden. Wenn die geplante TIPS-Anlage wegen eines niedrigen Druckgradienten < 10 mmHg abgebrochen wird, ist zu empfehlen, vor Beendigung des Eingriffs eine transjuguläre Leberbiopsie vorzunehmen, falls die Zirrhose noch nicht eindeutig nachgewiesen wurde.

Sehr großvolumige spontane portosystemische Kollateralen können den Druckgradienten reduzieren. In diesem Falle spricht ein Druckgradient < 12 mmHg nicht gegen eine TIPS-Anlage, wenn der „wahre“ Gradient nach Verschluss der Kollateralen über diesen Grenzwert liegt. Sedierung (Propofol) oder Hypovolämie (akute Blutung, großvolumige Parazentese) können auch zu einem falsch niedrigen Druckgradienten führen 153 . Bei Patienten, die innerhalb der Phase der akuten Blutung einen TIPS erhalten, kann eine Hypovolämie vorliegen, die sich auf den Druckgradienten auswirken kann 154 . Dasselbe kann für die Aszitespatienten gelten, die unter einer maximalen Diuretikatherapie stehen oder am Tage vor der TIPS-Anlage eine großvolumige Parazentese erhalten haben. Falls Patienten bei der Intervention einen sehr niedrigen Druckgradienten um 12 mmHg aufweisen, müssen diese Umstände berücksichtigt werden. Bei Patienten mit einer Varizenblutung sollte eine adjuvante Embolisation der Varizen durchgeführt werden 155 . Ebenso sollte bei Patienten mit Blutung aus Magenvarizen eine Embolisation der speisenden Kollateralen erfolgen, weil diese auch mit niedrigerem Druckgradienten bluten können 156 .

Der Druckgradient (üblicherweise HVPG) korreliert mit dem Risiko der Entwicklung von Symptomen der portalen Hypertension. Wenn der Gradient einen Grenzwert von 10 mmHg überschreitet, besteht das Risiko der Entwicklung von Varizen und ab Werten von > 12 mmHg steigt das Risiko von Varizenblutungen 158 159 163 164 . Die weitere Zunahme des HVPG auf > 20 mmHg hat einen deutlichen Effekt auf das 1-Jahres-Überleben (64 % vs. 20 % bei HVPG < 20 mmHg) 165 . In dieser Situation führte die frühe TIPS-Implantation zur Rezidivblutungsprophylaxe mit Senkung des Druckgradienten zu einem Überlebensvorteil 166 .

Bei Patienten mit Aszites ist die Situation nicht so eindeutig. Als klinisch-signifikante portale Hypertonie ist ein HVPG von 10 mmHg etabliert. Über diesem Wert ist das Risiko einer hydropischen Dekompensation deutlich erhöht, sodass eine Senkung auf bzw. unter diesen Wert als anstrebenswert anzusehen ist. Allerdings zeigen neuere Daten, dass eine Senkung über 60 % des PPG bei der TIPS-Anlage das Ansprechen nach TIPS und damit das Überleben deutlich verbessert 183 .

Die Druckmessungen bei der Intervention entsprechen nicht exakt den Drücken im weiteren Zeitverlauf. Zu den Effekten von Sedierung und Hypovolämie zum Zeitpunkt des Eingriffs kann eine Rückbildung der splanchnischen Vasodilatation im Verlauf hinzukommen 155 . Zusätzlich kann sich in den ersten Wochen und Monaten der Durchmesser des Stentgrafts vergrößern, was den Druck im Portalsystem weiter reduziert 184 . Daher wird die Einlage eines neueren „controlled expansion“ Stents empfohlen, bei dem die Selbstexpansion des Stentgrafts weitestgehend verhindert werden kann 177 .

Neben der kontinuierlichen Überwachung des Kreislaufs (Herzfrequenz, Blutdruck) und einer Sauerstoffsättigung (SpO _2_ ) sollte auch eine Analgesie und ggf. in ausgewählten Fällen eine Sedierung durchgeführt werden. In diesem Zusammenhang wird bezüglich apparativer und personeller Voraussetzung auf die Leitlinie der DGVS zur Sedierung in der Endoskopie verwiesen 185 . Geplante Eingriffe können in einer Kombination aus Benzodiazepin plus Opioid durchgeführt werden 185 . In Notfallsituationen, z. B. bei Varizenblutung, oder bei Risikopatienten (z. B. schwere Adipositas), ist eine Anlage unter Schutzintubation und entsprechender Vollnarkose empfehlenswert 156 . Zu beachten ist dabei, dass die Sedierungs- bzw. Narkosemedikamente möglicherweise den portalen Druck und damit die Messwerte bei der TIPS-Implantation verändern können. Dies ist insbesondere bei Propofol mit seiner depressiven Wirkung auf den Blutdruck bzw. peripheren Gefäßwiderstand und auf das Herzzeitvolumen zu erwarten 185 . Zudem hat Propofol nur einen minimalen analgetischen Effekt 185 . Tatsächlich konnte in einer Studie die Abnahme des portalen Drucks, sowohl bei der HVPG-Messung als auch bei der TIPS-Implantation, unter einer tiefen Propofol-Sedierung gezeigt werden 186 . Zur Wirkung einer Midazolam-Sedierung auf den portalen Druck existiert eine Untersuchung mit Messung des Hepatisch-venösen Druckgradienten 187 . Hierbei fanden die Autoren, dass eine niedrige Dosis von Midazolam den portalen Druck nicht verändert 187 .

### Rolle des Durchmessers des Stentgrafts

Bei der TIPS-Neuanlage sollten primär Stentgrafts mit variablem Durchmesser (bis max. 10 mm) eingesetzt werden. Der initiale effektive Durchmesser (typischerweise 6–8 mm) sollte mittels einer Patienten-individuellen Strategie in Abhängigkeit der individuellen Risikokonstellation (Kardiale Situation, frühere HE-Episoden etc.) angestrebt werden. Falls der Therapieeffekt ungenügend sein sollte, kann individuell bei der Implantation oder in einer anderen Sitzung nachdilatiert werden. Sofern individuelle Aspekte es erfordern, kann der Stentgraftdurchmesser größer als 8 mm gewählt werden.

### Embolisation der spontanen portosystemischen Kollateralen

Im Rahmen einer portalen Hypertension entstehen bei etwa 60 % der Patienten spontane porto-systemische Shunts, welche mit dem Vorhandensein einer hepatischen Enzephalopathie, einer erhöhten Rate an Komplikationen und einer erhöhten Letalität assoziiert sind 188 . Eine aktuelle Arbeit konnte zusätzlich die Bedeutung der Shuntgesamtfläche gegenüber der Beurteilung des größten Shuntdurchmessers herausarbeiten 189 . Auch hier zeigt sich eine Zunahme der hepatischen Enzephalopathie und der Letalität in Abhängigkeit der Shuntfläche 189 . Die relevanten Kollateralen im Rahmen der TIPS-Implantation bei Patienten mit portalem Hypertonus umfassen Varizen des Magens oder des Ösophagus, die Umbilikalvene und weitere portosystemische, v. a. splenorenale Shunts. Die Implantation eines TIPS vergrößert die Shuntgesamtfläche. Das Vorhandensein von Shunts bei der TIPS-Anlage ist ein Risikofaktor für das Entstehen von Kurzzeitkomplikationen (30 Tage) 190 . Vor diesem Hintergrund würde eine Embolisation von Varizen/Shunts die Gesamtfläche der Shunts verringern und könnte daher von Vorteil sein. Die Ergebnisse bezüglich der Embolisation der Shunts/Varizen während der TIPS-Implantation sind jedoch widersprüchlich. Eine deutsche prospektive Studie untersuchte die Embolisation der portosystemischen Shunts bei 95 Patienten mit Varizenblutung und TIPS-Implantation 191 . Die Embolisation wurde während der TIPS-Anlage in Abhängigkeit der Kontrastmittelfüllung der Varizen und des portosystemischen Druckgradienten über 12 mmHg durchgeführt 191 . Die Embolisationsgruppe zeigte eine signifikant niedrigere Reblutungsrate 191 . Eine neuere retrospektive Studie mit 101 Patienten fand ebenfalls eine signifikant niedrigere Reblutungsrate (18,8 % vs. 5,7 %) und eine geringere Enzephalopathierate in der Gruppe der Patienten mit Embolisation 192 , was kürzlich wieder in einer prospektiven und randomisierten Studie bestätigt wurde 193 . Demgegenüber fanden andere ältere Studien keine Unterschiede in der Letalität, der Enzephalopathierate, der TIPS-Revisonsrate und der Reblutungsrate 154 194 . Eine Metaanalyse aus dem Jahr 2014 zeigte einen möglichen Vorteil für die Gruppe der Patienten mit Embolisation in Bezug auf die Reblutungsrate, bei deutlicher Heterogenität der Studienpopulation in Bezug auf verwendete Stents, Typ der Varizen und des verwendeten Embolisats/Coils hervor 195 .

Aufgrund dieser Studienlage ist eine generelle Empfehlung bezüglich der Embolisation von Varizen/Shunts nicht möglich, erscheint aber bei Patienten mit portalhypertensiver Blutung als Indikation zur TIPS-Implantation durchaus primär sinnvoll 196 . Für die Embolisation können Metallspiralen/Coils (Magenvarizen und Ösophagusvarizen ( Abb. 3e )) oder n-Butyl-2-Cyanoacrylat (Ösophagusvarizen) verwendet werden 197 . Nur wenige vergleichende Studien bezüglich der zwei Methoden sind vorhanden 198 . Als Zeitpunkt der Embolisation erscheint die Embolisation vor Implantation der Stents sinnvoll 197 . Dieser Zeitpunkt hat ein minimiertes Risiko der Butyl-2-Cyanoacrylat-Abschwemmung durch den TIPS-Trakt, was nicht unerheblich ist (12,5 %). Auch nach der TIPS-Implantation kann eine zusätzliche Embolisation der Varizen vorgenommen werden, insbesondere wenn ein porto-systemischer Druckgradient über 12 mmHg bei Blutungsindikation (erhöhtes Reblutungsrisiko) vorliegt und/oder die Kollateralen trotz TIPS auch bei Aszites deutlich dargestellt werden 174 178 . Eine Embolisation der Varizen führt zu einer Veränderung des portalen Drucks, sodass nach Embolisation eine genauere Einstellung des porto-systemischen Druckgradienten erfolgen kann 197 .

## Nachsorge der Patienten mit TIPS

### Postinterventionelles Management

Postinterventionelle Komplikationen nach TIPS-Anlage ( Tab. 2 ) werden in Studien mit großer Fallzahl in der Regel nach Schweregrad und Dauer der Therapie in gering (minor: keine Konsequenz oder Observation über Nacht) oder schwer (major: Überwachung und Therapie 48 Stunden oder länger) unterteilt. Schwerwiegende Komplikationen treten in ca. 3 % der Fälle auf und führen bei 1 % der Patienten zu einer erhöhten interventionsbedingten Letalität innerhalb von 30 Tagen 156 166 . Die Mehrheit der Todesfälle ist mit präexistierenden Faktoren für einen ungünstigen post-interventionellen Verlauf nach TIPS assoziiert wie einem hohen MELD- oder Child-Pugh-Score und kann daher durch eine sorgfältige Patientenauswahl beeinflusst werden 199 . Im Gegensatz zur 90-Tage-Letalität scheint ein höheres Alter > 65 Jahre keinen Einfluss auf die peri-interventionelle Komplikationsrate zu haben 124 .

**Tab. 2:** Peri-interventionelle Komplikationen modifiziert nach 166 .

Art der Komplikation	Häufigkeit (%)
***Primär interventionell***	
**Hämatoperitoneum**	0,5
**Biliäre Peritonitis**	1
**Hämobilie**	2
**Verletzung der hepatischen Arterie**	1
**Hepatischer Infarkt**	0,5
**Fehlerhafte Position des Stents**	1
**Lokale Blutung Einstichstelle**	2
**Strahlungsbedingte Hautverbrennung**	0,1
***Sekundär organbezogen***	
**Enzephalopathie**	15–25
**Fulminantes Leberversagen**	1
**Akutes Nierenversagen mit Dialyse**	0,25
**Passageres Nierenversagen**	2
**Pulmonales Ödem**	1
**Fieber**	2

Klassifiziert werden können die Komplikationen als unmittelbare Folge der Intervention oder als sekundäres Versagen eines spezifischen Organs 167 . Unter den primär interventionellen Komplikationen werden demnach Blutungen und Leckagen insbesondere in das Peritoneum oder das biliäre System sowie Gefäßverletzungen zusammengefasst 200 . Als potenziell letaler Notfall gilt außerdem die fehlerhafte Positionierung oder Migration des Stents, da diese in der Vergangenheit in Einzelfällen zu Gefäßperforationen geführt haben. Die Entwicklung neuerer ummantelter Stents mit spezifischer Positionierungshilfe konnte jedoch sowohl die Gefahr einer Fehlpositionierung als auch die Häufigkeit von Leckagen und Blutungen signifikant reduzieren 201 . Die postinterventionelle Überwachung entspricht nach Einsatz von Sedierung den Vorgaben der DGVS-Leitlinie zur Sedierung in der Endoskopie 185 . Nach der Intervention ist eine Überwachung mindestens zeitweise anzustreben. Am Tag nach der Intervention ist eine Bestimmung der Transaminasen, der Cholestaseparameter, inklusive Bilirubinwerte, der Gerinnungsparameter und der kleinen Blutbildparameter empfohlen. Hintergrund der Bestimmung der Laborparameter ist die frühzeitige Erkennung von möglichen Komplikationen, insbesondere von prozedurbedingten Komplikationen wie etwa Ischämie-bedingter Leberschädigungen.

Eine Metaanalyse konnte im Jahr 2023 zwar zeigen, dass die TIPS-Implantation das Risiko einer weiteren Dekompensation senkt 202 , trotzdem können prinzipiell nach TIPS-Implantation, Organdysfunktion und Organversagen bis zum Akut-auf-chronisches Leberversagen auftreten, welche in der Regel durch eine Vorschädigung zumindest begünstigt werden 156 203 . Typischerweise ist hier die eine subklinische Kardiomyopathie zu erwähnen 126 . Auch eine zunehmend abnehmende Leberschädigung (Transaminasen, Bilirubin-Anstieg) und/oder schwere HE-Episoden können vor allem bei Hochrisikopatienten (höheres Bilirubin, MELD, Alter, etc.) auftreten 203 . Bei letzterem, könnte ein konkurrierender Shunt ursächlich sein und wäre therapeutisch mit einer Reduktion des Shunt-Volumens durch die Embolisation der Umgehungskreisläufe favorisiert, und dann eine TIPS-Reduktion diskutiert. Parallel dazu sollte eine Lebertransplantation diskutiert werden 156 .

Die Messung des portosystemischen Druckgradienten erfolgt in der Regel als Erfolgskontrolle unmittelbar nach Anlage des portosystemischen Shunts noch als Teil der Prozedur 156 200 .

Neben dem klinischen Erfolg der TIPS-Anlage (keine neue Episode einer Varizenblutung bzw. keine Parazentese) wird die TIPS-Funktion mit einer sonografischen Kontrolle der Flussgeschwindigkeit der Pfortader (≥ 30 cm/s) und im Stent (≥ 40 cm/s) überprüft 166 204 . Trotz der eher geringeren Sensitivität der Methode kann der Ultraschall als initiale Screening-Methode zur Detektion einer Stentdysfunktion dienen. Insbesondere bei nicht-beschichteten Stents kommt es durch Thrombosen und Hyperplasie der Intima innerhalb der ersten zwei Jahre in über 50 % der Fälle zum Verschluss, während beschichtete Stents im gleichen Zeitraum in über 75 % offen bleiben 148 . Trotzdem kann es bei allen Stents zur Dysfunktion kommen, welche sowohl bei einer Flussgeschwindigkeit-Verlangsamung als auch segmentale Beschleunigung in der Duplex-Sonografie vermutet werden sollen. Aufgrund der akustischen Barriere der Ummantelung sind diese aber auch technisch in der Sonografie schwerer zu beurteilen, vor allem die distale Flussgeschwindigkeit ist innerhalb der ersten 14 Tage nur schlecht abzuleiten 204 . Die Autoren empfehlen weitere sonografische Kontrollen alle 6 Monate, ein Vorgehen, das sich mit dem Screening für die Entwicklung eines hepatozellulären Karzinoms (HCC) deckt 205 . Eine invasive Darstellung des TIPS inklusive Messung des Pfortaderdrucks wird bei auffälligen oder unklaren Ultraschallbefunden sowie bei fehlendem klinischem Erfolg empfohlen 206 . Der klinische Erfolg zeigt sich bei der Indikation Varizenblutung durch das Fehlen einer frühen Rezidivblutung und bei der Indikation Aszites das Fehlen von Aszites (bzw. punktablen Aszites) innerhalb von 6 Wochen. Deshalb wäre eine ambulante Vorstellung in 4–6 Wochen, dann nach 3 Monaten und anschließend alle 6 Monate nach TIPS zu empfehlen.

### Untersuchungen bei der Wiedervorstellung

#### Erkennung der TIPS-Dysfunktion

Mit der Einführung von PTFE-beschichteten Stents konnten die Raten an TIPS-Dysfunktionen im Vergleich zu unbeschichteten Stents reduziert werden (25 % vs. 50 % in den ersten zwei Jahre 148 ). Die Doppler-Sonografie ist für die Darstellung der Perfusion der Pfortaderäste und des TIPS-Traktes (siehe oben) geeignet. Zudem kann sie eingesetzt werden, um prozedurale Komplikationen vor Entlassung der Patienten zu detektieren 204 . Die meisten Patienten, die mit einer TIPS-Anlage behandelt werden, sind Patienten mit Leberzirrhose. Eine Doppler-Sonografie kann daher im Rahmen der empfohlenen TIPS-Kontrollen (siehe oben) und halbjährlichen Screenings für hepatozelluläre Karzinome erfolgen 207 . Diese regelmäßigen sonografischen Untersuchungen eignen sich auch, um das klinische Ansprechen bei refraktärem Aszites zu beurteilen. Routinemäßige invasive transjuguläre Darstellungen des TIPS-Traktes in Durchleuchtung sind außer bei Doppler-sonografischem oder klinischem Verdacht auf TIPS-Dysfunktionen nicht empfohlen. Das Überprüfen des Körpergewichts (ggf. periphere Ödeme) kann eine mögliche TIPS-Dysfunktion identifizieren.

#### Diagnostik der hepatischen Enzephalopathie

Trotz sorgfältiger Patientenauswahl ist das Risiko für das Auftreten von Episoden hepatischer Enzephalopathie nach Implantation eines TIPS erhöht. Dennoch ist die Studienlage zur Inzidenz von HE nach TIPS-Anlage eingeschränkt. Die Inzidenz von post-TIPS-HE schwankt zwischen 15 und 67 % in 2 Jahren. Die Inzidenz von persistierender klinisch-manifesten HE liegt bei 8 % und die von de-novo nicht klinisch-manifesten HE bei etwa einem Drittel der Patienten 8 14 132 208 209 210 211 212 213 . Neuropsychometrische Untersuchungen sind insbesondere bei der Diagnose von nicht klinisch-manifesten HE hilfreich. Hierbei zeigen Patienten bestimmte, reversible, quantifizierbare neuropsychologische oder elektroenzephalografische Auffälligkeiten 214 . Dies ist insbesondere wichtig, da das Vorhandensein einer nicht klinisch-manifesten HE mit der Entwicklung von klinisch-manifesten HE assoziiert ist 215 .

Die aktuelle deutsche Leitlinie empfiehlt den Einsatz neuropsychometrischer Tests zur Diagnose von nicht klinisch-manifesten HE. Daten zu den optimalen Zeitpunkten zum Screening auf nicht klinisch-manifesten HE nach TIPS-Anlage liegen nicht vor. Aufgrund der erhöhten Raten von HE innerhalb der ersten 2 Jahre scheint ein frühes Screening, z. B. vor stationärer Entlassung und im Rahmen der regelmäßigen Nachsorge alle 6 Monate, sinnvoll. Das Vorhandensein einer klinisch-manifesten HE wird entsprechend der Leitlinien klinisch anhand der West-Haven-Kriterien diagnostiziert. Die West-Haven-Kriterien sind das am häufigsten angewandte Graduierungssystem zur Beurteilung der HE bei Patienten mit Leberzirrhose und zeichnet sich durch hohe Praktikabilität im klinischen Alltag aus 136 216 217 .

#### Diagnostik der kardialen Belastung

Ein TIPS greift deutlich in die portalvenöse und systemische Hämodynamik sowie die kardiale Funktion von Patienten mit Leberzirrhose ein 218 219 220 221 222 . Die Datenlage zur Inzidenz von symptomatischer Herzinsuffizienz nach TIPS ist uneinheitlich. Eine retrospektive Studie beschrieb lediglich eine symptomatische Herzinsuffizienzrate von 0,9 % nach TIPS 223 . Allerdings beschreibt eine prospektive Studie aus Frankreich die Entwicklung einer Herzinsuffizienz nach TIPS-Anlage bei 20 % der Patienten innerhalb eines Jahres 224 . Bei diesen Patienten war die Sterblichkeit mit 25 % hoch. Eine prospektive Fall-Kontroll-Studie zeigte, dass auf 8 mm dilatierte Controlled Expansion Stents eine niedrigere Rate an Hospitalisierung wegen kardialer Dekompensationen aufwiesen, verglichen mit herkömmlichen Stents 177 198 . Studien, die einen Benefit durch eine solche kardiale Evaluation und den optimalen Zeitpunkt nach TIPS untersuchen, liegen nicht vor, sodass noch keine Empfehlung abgegeben werden kann. Einige früh auftretende hämodynamische und kardiale Veränderungen scheinen sich innerhalb der ersten Wochen nach TIPS wieder zurückzubilden 219 . Signifikante Veränderungen mit prognostischer Bedeutung können jedoch schon 6 Wochen nach TIPS mit speziellen Echokardiografietechniken (Speckle-Tracking) nachgewiesen werden 222 .

### Antikoagulation Post-TIPS

Trotz hoher technischer Erfolgsraten der TIPS-Anlage von > 90 % 2 148 225 wird die langfristige Offenheitsrate durch akute wie auch chronische Stentthrombosen sowie in geringerem Maß auch eine endotheliale Hyperplasie bzw. eine Kombination beider Prozesse limitiert. Eine externe Kompression des TIPS-Traktes bei fortschreitender Leberzirrhose wird als weitere potenzielle Ursache für Restenosen und TIPS-Dysfunktion diskutiert, ohne dass hierfür eine klare Evidenz existiert. Die Einführung von Polytetrafluoroethylene (PTFE)-Stentgrafts hat im Vergleich zu konventionellen Nitinol-Stents die Offenheitsrate sowohl in nicht randomisierten Fallserien 225 226 227 wie auch in prospektiv randomisierten Studien 148 232 signifikant verbessert. In der prospektiv randomisierten Arbeit von Bureau et al wurde eine primäre Offenheitsrate PTFE-gecoverter TIPS-Stents von 86 % nach 1 Jahr und 76–80 % nach 2 Jahren erreicht 228 . Aktuellere Studien berichten Offenheitsraten von über 90 % 225 229 , was in zwei größeren Metaanalysen bestätigt werden konnte 150 174 . Während verschiedenste Regime der Antikoagulation und Thrombozytenfunktionshemmung nach Stent-Implantation in den Koronararterien sehr gut untersucht sind, gibt es keine validen Daten für den Einsatz von Antikoagulantien und Plättchenhemmung nach TIPS-Anlage. Auch gibt es kaum gesicherte Kenntnisse über die Wirkung von Antikoagulantien wie auch Thrombozytenfunktionshemmern im portalvenösen System. Bis dato gibt es nur vereinzelte Berichte über einen möglichen Benefit einer gezielten Antikoagulation sowie Thrombozytenfunktionshemmung nach TIPS 231 232 ), die zudem aus der Ära nicht gecoverter Nitinol-Stents stammen und deren Ergebnisse nicht auf PTFE-gecoverte TIPS-Stents interpoliert werden können.

Bisher wurden fortgeschrittene Stadien der Leberzirrhose sowie eingeschränkte Leberfunktion eher mit einer Hypokoagulation aufgrund einer verminderten Synthese von Gerinnungsfaktoren assoziiert. Dieses Paradigma wird aktuell zunehmend in Frage gestellt. Mehrere Studien konnten zeigen, dass der reduzierten Synthese gerinnungshemmender Faktoren die verminderte Synthese von gerinnungsfördernden Faktoren gegenübersteht, mit einem entsprechend erhöhtem Risiko für thrombembolische Ereignisse 233 . Vor dem Hintergrund der komplexen Gerinnungsalteration in Stadien fortgeschrittener Leberzirrhose ist sowohl die grundsätzliche Wirkung, wie auch die klinische Notwendigkeit einer Antikoagulation oder Thrombozytenfunktionshemmung nach TIPS-Anlage unklar. Einzelne Retrospektive Analysen haben einen Überlebensvorteil für die Gabe von ASS nach TIPS-Anlage ergeben, was zum einen kontrovers diskutiert wird, zum anderen der Effekt mutmaßlich nicht auf eine verbesserte Offenheitsrate des TIPS zurückzuführen ist, sondern auf eine Reduktion der hepatischen Inflammation und einen langsameren Progress der zugrunde liegenden Lebererkrankung 234 235 .

Zusammenfassend existiert aktuell kaum Evidenz für oder gegen den Einsatz von Antikoagulation oder Thrombozytenfunktionshemmmern im Rahmen von TIPS, weder die EASL noch die AASLD geben hierzu eine Empfehlung ab, außer bei vaskulären Lebererkrankungen und prothrombotischen Ereignissen.

### Medikation nach TIPS

#### Vorbeugung der hepatischen Enzephalopathie

Bei Patienten mit post-TIPS HE konzentriert sich die Behandlung auf die Identifizierung und Korrektur des auslösenden Ereignisses, die allgemeinen Lebensstilempfehlungen und ein angemessenes Ernährungsmanagement 236 . Eine randomisierte kontrollierte Studie aus dem Jahr 2005 zeigte, dass die Prophylaxe von Laktulose oder Rifaximin im ersten Monat bei hepatischer Enzephalopathie nach TIPS nicht sehr wirksam zu sein scheint 212 . In ähnlicher Weise wurde auch festgestellt, dass die Albumininfusion keine Rolle bei der Prävention von HE nach TIPS spielt 237 . Eine kürzlich durchgeführte RCT zeigte jedoch, dass eine bereits vor TIPS begonnene Rifaximin-Therapie gut vertragen wurde und sich das Risiko für klinisch-manifeste HE bei Patienten mit Zirrhose, die mit TIPS behandelt wurden, innerhalb von 6 Monaten verringerte 238 . Rifaximin sollte daher für die Prophylaxe von post-TIPS-HE im off-label-Einsatz in Betracht gezogen werden. Darüber hinaus wurde in einer retrospektiven Studie über die Verwendung von Protonenpumpenhemmern (PPI) berichtet, die mit einem erhöhten Risiko für neue oder sich verschlechternde HE nach TIPS-Anlage verbunden waren 239 . Eine strenge Indikationsprüfung sollte daher bei bestehender PPI-Therapie erfolgen.

#### Die Behandlung mit nicht-selektiven Betablockern

Es existieren Daten, die zeigen, dass die Weitergabe eines nicht-selektiven Betablockers nach TIPS-Anlage einen stärkeren Effekt auf die Pfortaderdrucksenkung hat 240 . Inwieweit eine Weitergabe des Betablockers die Komplikationsrate des TIPS im Verlauf günstig beeinflusst, ist trotzdem unklar. Bei Nebenwirkungen des Betablockers sollte diese Medikation ausgeschlichen werden.

#### Medikation der renalen Dysfunktion

Das Grundprinzip des TIPS besteht hauptsächlich darin, den Portaldruck zu verringern und das effektive Blutvolumen und damit die Nierenperfusion zu erhöhen. Etwa vier Wochen nach der TIPS-Implantation verbessert sich im Allgemeinen die Natriumausscheidung und die Nierenfunktion 241 242 . Der Aszites wird unter diesen Bedingungen wieder Diuretika-sensibel und kann dann entsprechend des Ausmaßes der Aszitesbildung behandelt werden. Der Anteil der Patienten, die nach TIPS-Implantation eine komplette Remission erlangen, also ein vollständiges Verschwinden des Aszites, steigt mit der Zeit an, von 54,1 % nach 12 Monaten auf 77,6 % nach 24 Monaten 172 . Als Ursache hierfür müssen die langsame Adaptation des Kreislaufs an die neuen hämodynamischen Verhältnisse angesehen werden 243 . Eine retrospektive Analyse konnte zeigen, dass persistierender Aszites nach TIPS-Implantation ein unabhängiger Risikofaktor für die Notwendigkeit einer Lebertransplantation oder das Versterben ist 244 .

### TIPS-Dysfunktion

Eine TIPS-Dysfunktion ist definiert als ein Verlust der Dekompression des Portalvenensystems aufgrund eines Verschlusses oder einer Stenose des TIPS 245 . Eine Stenose von 50 % wird häufig als Kriterium für die angiografische TIPS-Dysfunktion verwendet, obwohl keine genaue Definition vorliegt. Darüber hinaus weist ein Anstieg des portosystemischen Druckgradienten über 10 mmHg oder ein Wiederauftreten der Komplikation der portalen Hypertonie auf eine TIPS-Dysfunktion hin 228 246 .

Als Screeningmethode ist die Duplexsonografie geeignet. In einer aktuellen Metaanalyse zur Ultraschalldiagnostik der TIPS-Dysfunktion beträgt die Sensitivität 0,82 bei geringer Spezifität von 0,58 247 . Die Sensitivität und Spezifität für TIPS-Verschlüsse beträgt hingegen 0,96 und 1. Mittels Duplexsonografie kann die Flussgeschwindigkeit innerhalb des TIPS-Traktes und in den vorgeschalteten Abschnitten der Pfortader und der nachgeschalteten Lebervene gemessen werden. Im Falle einer TIPS-Stenose finden sich erhöhte oder erniedrigte Flussgeschwindigkeiten innerhalb des Shunts und ggf. eine erniedrigte Flussgeschwindigkeit im Pfortaderstamm. Typische Dysfunktionszeichen sind eine Reduktion der Flussgeschwindigkeit im Pfortaderhauptstamm (Senkung um 30–50 % oder unter 30 cm/s). Zudem sind eine Flussrichtungsumkehr (hepatopetal im Pfortaderast, wo nicht der TIPS liegt) und eine segmentale Flussbeschleunigung im TIPS (> 50–80 %) Hinweise für eine TIPS-Dysfunktion. Auch das (Wieder-) Auftreten von Aszites ist ein Zeichen einer TIPS-Dysfunktion.

Bei Hinweisen für eine Dysfunktion, sollte eine kontrastverstärkte portal-venöse CT-Angiografie zur anatomischen Darstellung der porto-venösen Gefäßachse oder eine kontrastverstärkte portal-venöse MR-Angiografie als Alternative zur Anwendung kommen. Im Vergleich zur CT-Angiografie ist der intraluminale Anteil des TIPS-Shunts bzw. des Stentgrafts in der Regel durch Metallartefakte weniger gut beurteilbar. Entsprechende Stenosen sind oft nicht ausreichend quantifizierbar, hochgradige Stenosen innerhalb des Stentgrafts können nicht mit ausreichender Sicherheit gegenüber Verschlüssen abgegrenzt werden. Die zuverlässigste Diagnose einer TIPS-Dysfunktion ergibt sich durch eine invasive Messung des porto-systemischen Druckgradienten.

#### Erweiterung des TIPS-Traktes

Bei klinischen Zeichen der persistierenden portalen Hypertension (wie z. B. therapierefraktärer Aszites oder neuerliche Blutungen aus Oesophagusvarizen) empfiehlt sich eine interventionelle TIPS-Kontrolle. Hierbei sollte der portosystemische Druckgradient bei Blutungen unter 12 mmHg gesenkt werden, optimal zwischen 8 und 10 mmHg 248 . Bei therapierefraktärem Aszites ist der optimale portosystemische Druckgradient weniger exakt definiert 249 . Druckgradienten von weniger als 8 mmHg gehen mit einer erhöhten Gefahr der hepatischen Enzephalopathie einher 250 .

In aller Regel ist eine einfache PTA des TIPS-Traktes mit einem Ballonkatheter unter Zuhilfenahme einer PTA-Druckspritze ausreichend. Eine zusätzliche Stentimplantation kann jedoch notwendig werden, bspw. wenn sich ausgeprägte Knickbildungen zeigen, oder der initiale TIPS zu kurz gewählt wurde und den Parenchym-Trakt meist lebervenennah nicht vollständig überstentet 251 . Für den Fall, dass der initiale TIPS der kranialen Lebervene aufsteht und sich dadurch ein funktionelles Ausflusshindernis ergibt ( Abb. 3f–h ), ist die Implantation eines ungecoverten Stents in aller Regel ausreichend, allerdings sollte die adäquate Senkung des Druckes nicht eine LTX gefährden (z. B. Malposition).

#### Revaskularisation bei TIPS-Okklusion

Bei einer kompletten Okklusion des TIPS-Traktes sind mechanische Thrombektomien oder eine kathetergestützte Thrombolyse mit und ohne Stentimplantation mögliche Herangehensweisen 252 . Bei Versagen des transjugulären Zugangsweges finden der translienale Zugang Anwendung 253 . Die technische Erfolgsrate wird in Publikationen mit über 90 % angegeben 254 . Beim transsplenischen Zugangsweg wird sonografisch eine Chiba-Nadel in eine intrasplenische Vene eingebracht, der Draht über einen Snare-Katheter eingefangen und transjugulär ausgeleitet, um die weiteren Arbeitsschritte von transjugulär zu ermöglichen 255 . Größere Fallserien zu diesem Zugangsweg sind derzeit jedoch noch nicht publiziert. Scheitern alle Versuche, den originären TIPS-Trakt zu revaskularisieren, sollte die Möglichkeit einer parallelen TIPS-Neueinbringung evaluiert werden 256 . Hierbei soll dann eine Antikoagulation zumindestens für 6 Monate analog zu der Pfortaderthrombose erfolgen.

#### TIPS-Reduktion

**Abb. 5:**
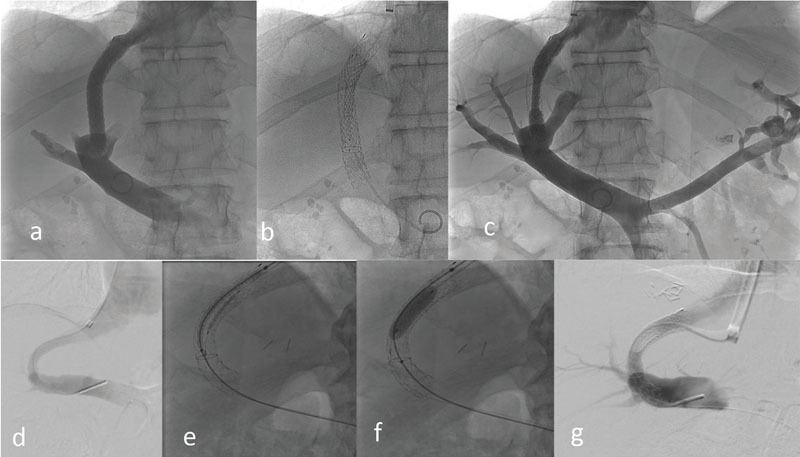
Die obere Bildreihe zeigt eine TIPS-Reduktion mittlels „Sheath control technique“ die untere mittels Doppel-Stent-Technik ( **a–c** ). **a** Überdrainage des eingebrachten TIPS ohne Rest-Kontrastierung der Pfortaderäste ( **d** ). Parallele Platzierung eines selbstexpandierbaren Stentgrafts und eines ballonexpandierbaren Stents ( **e** ). Applikation der beiden Stents ( **f** ). Deutlich verbesserter Fluss auf den Pfortaderästen mit KM-Aussparung im TIPS-Trakt als Ausdruck der Lumenreduktion ( **g** ). [rerif]

Bei persistierenden Episoden schwerer hepatischer Enzephalopathie (Nardelli et al. empfehlen 3 Episoden schwerer Enzephalopathie innerhalb von 3 Monaten) 257 kann es notwendig werden, den TIPS-Fluss zu reduzieren ( Abb. 5a–c ). Seltener können auch Leberversagen oder eine kardiale Dekompensation bzw. Zeichen der zunehmenden Rechtsherzbelastung hierfür ursächlich sein. In Einzelfällen können Perfusionsstörungen des Leberparenchyms unterschiedlicher Ausprägung eintreten, von vorübergehenden Transaminasenerhöhungen bis hin zur manifesten Leberischämie mit strukturellen Parenchymveränderungen reichend (z. B. Lebernekrosen). Lebernekrosen können insbesondere eintreten, wenn eine gleichzeitige Beeinträchtigung der arteriellen Perfusion besteht. Daher sollte bei der Indikationsstellung geprüft werden, dass die arterielle Perfusion unbeeinträchtigt ist 258 259 260 .

Zur Lumenreduktion stand in der Vergangenheit ein spezieller Reduktionsstent zur Verfügung 261 . Dieser wurde jedoch mittlerweile vom Markt genommen und ist nicht mehr erhältlich. Daher muss auf alternative Methoden zurückgegriffen werden. Diese existieren in Form von Einzelfallberichten und Fallserien und sollten erfahrenen Anwendern vorbehalten bleiben 262 263 ( Abb. 5d–g ).

### Therapeutische Optionen bei portal-venöser Blutung nach TIPS

Im Falle einer gastrointestinalen Blutung nach TIPS sollte TIPS-Offenheit geprüft und dann ggf. der TIPS dilatiert werden. In Fällen, in denen eine Erweiterung des TIPS nicht möglich ist, wurde in Einzelfällen auch eine zweite parallele TIPS-Anlage mit gutem Therapieerfolg durchgeführt 256 .

Falls eine Dilatation nicht möglich ist, sollte der interventionelle Verschluss von Varizen mittels Coiling über den TIPS-Zugang erfolgen. In einem kleinen Kollektiv von Patienten nach Lebertransplantation (n = 18) konnte hier bei 82 % der Patienten eine erfolgreiche Blutungskontrolle erreicht werden 264 . Eine prospektive Studie an 122 Patienten aus China konnte zeigen, dass die Kombination aus TIPS und zusätzlicher Embolisation der. V. coronaria ventriculi das Auftreten einer erneuten Blutung von 41,5 % auf 19,5 % reduzieren konnte 265 .

Ebenso möglich ist ein interventioneller Verschluss der Varizen über einen spontanen portosystemischen Shunt. Der sogenannte Ballon-okkludierende retrograde transvenöse Verschluss (BRTO) basiert auf einer erhöhten Inzidenz (60–85 %) von gastrorenalen Shunts 266 267 268 . Die Erfolgsrate dieser Intervention wird mit 79–100 % beschrieben bei einer Re-Blutungsrate von 0–14 % 269 . In einer retrospektiven Analyse aus Korea zeigte sich die BRTO in der Kontrolle von gastrischen Varizen sogar erfolgreicher als der TIPS (19,8 % vs. 8,6 % nach einem Jahr) 270 .

Coiling von Varizen auch in Kombination mit Histoacryl ist auch endosonografisch gesteuert möglich. Hier konnte in einem Kollektiv von 152 Patienten ein kompletter Verschluss der Varize bei 93 % der Patienten gezeigt werden. Zu einem Blutungsereignis kam es im Verlauf bei 3 Patienten, wobei der Anteil an Patienten mit aktiver Blutung mit 5 % sehr klein war. Bei 69 % der Patienten kam es kurz zuvor zu einem Blutungsereignis 271 .

Als weitere mögliche Intervention ist die transarterielle Milz(-teil)embolisation zu nennen. Interventionell kann eine zusätzliche Senkung des portalvenösen Drucks über eine zentrale oder periphere (Teil-)Partikelembolisation des Milzparenchyms erfolgen. Während die zentrale Milzarterienembolisation mit hoher Sicherheit durchgeführt werden kann, aber den portalvenösen Druck meist auch nur gering senkt, ist die periphere Milzarterienembolisation mit einem signifikanten Interventionsrisiko verbunden wie Milzinfarkte, septische Embolien und Pleuraergüsse. Das Embolisationsvolumen sollte nicht 50–70 % des Milzvolumens überschreiten 272 . Eine kleine retrospektive Fallserie konnte bei Patienten, die nicht für einen TIPS in Frage kamen, im Beobachtungszeitraum von 159 Monaten keine einzige erneute Blutung verzeichnen 273 .

Eine Cochrane-Analyse aus dem Jahr 2018 untersuchte 4 klinische Studien, in denen die Komplikationsraten und Effektivität einer chirurgischen Shunt-Anlage und eines TIPS verglichen wurde 274 . Nach Analyse von insgesamt 496 Patienten zeigte sich eine leicht niedrigere Komplikationsrate in Bezug auf Re-Blutung und eine Verbesserte 5-Jahres-Letalität 275 . Falls eine chirurgische Shunt-Anlage nicht möglich ist, wäre eine Sperr-Operation die Ultima Ratio.

### Therapeutische Optionen bei Persistenz des Aszites/Hydrothorax nach TIPS

Bei fortbestehendem Aszites sollte die Therapie neben einer TIPS-Revision, zunächst konservativ entsprechend den aktuellen Leitlinien u. a. durch eiweißreiche Ernährung und diuretischer Therapie erfolgen 34 136 .

Studien, die im Falle von persistierendem Aszites nach TIPS-Anlage ein dezidiertes Vorgehen beschreiben, existieren derzeit nicht. Die Therapieempfehlungen in diesen Fällen sollten sich demensprechend an den Studien orientieren, die an Patienten vor TIPS-Anlage oder nach Lebertransplantation durchgeführt wurden.

Die Implantation einer automatisierten low-flow-Aszitespumpe (AlphaPump – derzeit in Europa nicht erhältlich), welche den Aszites in die Harnblase kontinuierlich ableitet, konnte ebenfalls als Therapieoption bei therapierefraktärem Aszites etabliert werden. In einer größeren multizentrischen Analyse (n = 56) konnte eine Reduktion der Parazentesefrequenz von 2,17 auf 0,17 pro Monat erreicht werden 275 . In einer weiteren Meta-Analyse aus einer kontrollierten Studie und 8 Fallserien konnte eine mediane Reduktion der Parazentesefrequenz von 2,1 gezeigt werden 276 . Einzelne unveröffentlichte Fallberichte existieren mit adäquatem Therapieansprechen bei Implantation einer Aszitespumpe bei TIPS-Dysfunktion. Eine Kontraindikation zur Transplantation besteht nach Implantation einer solchen Pumpe nicht.

Als weitere interventionelle Maßnahme steht auch hier die Milzembolisation zur Verfügung. Die existierende Datenlage bezüglich des persistierenden Aszites beschränkt sich jedoch weitestgehend auf die zentrale Milzarterienembolisation bei Patienten im Rahmen der Lebertransplantation und die Fallzahlen sind insgesamt gering (n = 6 und n = 2) 277 278 . In einer weiteren Studie, in der insgesamt 10 Patienten mittels partieller Milzembolisation behandelt und analysiert wurden, konnte bei zwei Patienten mit Leberzirrhose vor Lebertransplantation ein vollständiges Therapieansprechen erzielt werden 279 .

Hinsichtlich des persistierenden hepatischen Hydrothorax nach TIPS-Anlage existiert eine sehr dünne Datenlage. Die Erfolgsrate eines TIPS bei hepatischem Hydrothorax ist mit ca. 60 % beschrieben 23 280 . Im Falle eines mangelnden Therapieerfolgs bleibt analog zur Behandlung des therapierefraktären Aszites die weitere konservative Therapie oder, wenn möglich, die Lebertransplantation. Kleine Fallserien mit 50 Patienten berichten über einen möglichen Therapieerfolg durch Pleurodese, wobei in den genannten Fallserien sich die Erfolgsraten je nach klinischem Zustand und unterschiedlicher Methode zwischen 37,5 % (Talkum oder Videoassistierte Thorakoskopie) 281 und 100 % (Povidin-Iod) unterscheiden 282 . Ein deutlich verbessertes Therapieansprechen zeigt sich, wenn im Rahmen des operativen Eingriffs die zugrunde liegende Zwerchfelllücke geschlossen werden kann 288 . Andere Fallserien berichten im Rahmen selbiger Interventionen jedoch Letalitätsraten von bis zu 45 % 283 . Eine zusätzliche Therapieoption bietet die Milzarterienembolisation (s. o.). Daten bezüglich des Therapieerfolgs dieser Maßnahme bei hepatischem Hydrothorax gibt es jedoch nicht.

### Management von Komplikationen nach TIPS-Anlage

#### EndoTIPSitis

Anhaltende Bakteriämien, die mit einer endovaskulären Infektion des TIPS-Stents assoziiert sind (EndoTIPSitis), sind seltene, aber schwere Komplikationen der TIPS-Anlage 285 . Der Begriff der EndoTIPSitis wurde 1998 eingeführt 286 . Eine konsentierte Definition der EndoTIPSitis existiert nicht. Die gängigste Definition wurde von Mizrahi et al. zusammengefasst 287 :

*Definitive Infektion: klinisch signifikante kontinuierliche Bakteriämie (Fieber und mehrere positive Blutkulturen) mit Vegetationen oder Thrombus innerhalb des TIPS.*

*Wahrscheinliche Infektion: Anhaltende Bakteriämie und persistierendes Fieber bei Patienten mit patentem TIPS nach Ausschluss anderweitiger Infektionsquellen.*

Einige Befundkonstellationen geben Verdachtsmomente für eine EndoTIPSitis. Quantitative Unterschiede in Kolonie-bildenden Bakterieneinheiten in Blutkulturen aus dem portalvenösen TIPS-nahen System und peripherem Blut deuten auf eine TIPS-assoziierte Infektion hin 288 289 . Eine TIPS-Stenose, -Thrombose oder -Vegetationen sind ebenfalls Risikofaktoren für eine EndoTIPSitis und in etwa der Hälfte der publizierten Fälle zu finden 287 290 . Letztlich scheint der Einsatz von mehreren überlappenden Stents ein Risikofaktor zu sein 285 .

Mikrobiologisch können eine Reihe verschiedener Erreger einer EndoTIPSitis zugrunde liegen. Die häufigsten Erreger sind Staphylokokken und andere Gram-positive Erreger sowie Enterokokken 291 292 293 294 295 296 297 298 .

Die Therapie der EndoTIPSitis (siehe Box 9 für die Diagnose) basiert auf der medikamentösen, antiinfektiven Therapie. Bei Verdacht auf eine Endotipsitis soll sofort die empirische antiinfektive Therapie mit Breitspektrum-Antiinfektiva erfolgen, die sowohl Gram-positive als auch Gram-negative Erreger miterfasst. Die gezielte Anpassung der antiinfektiven Therapie erfolgt dann entsprechend den nachgewiesenen Erregern in den Blutkulturen. Die mittlere Dauer der antiinfektiven Therapie der publizierten Fälle lag bei 30 Tagen (5–1460 Tage). Bei den erfolgreich behandelten Patienten (zwei Drittel) lag die mediane Behandlungsdauer bei 6 Wochen (2–101 Wochen). Zwar ergibt sich aufgrund der kleinen Fallzahlen keine Korrelation von Dauer der antiinfektiven Therapie und dem Behandlungserfolg, jedoch erscheint eine intravenöse Therapie (analog anderer endovaskulärer Infektionen) für 6 Wochen sinnvoll. In einem Drittel der Patienten ist die medikamentöse Therapie nicht in der Lage, die Infektion zu kontrollieren, was mit einer stark erhöhten Letalität einhergeht 287 299 300 . Insbesondere in seltenen Fällen von Pilzinfektionen scheint die Prognose ungünstig zu sein 299 300 . In solchen Patienten mit persistierender Bakteriämie trotz antibiogramm-gerechter antiinfektiver Therapie sollte eine Lebertransplantation evaluiert werden 301 302 303 304 .

#### Segmentale intrahepatische Cholestase

Eine kompressionsbedingt segmentale mechanische Cholestase durch den TIPS-Stent ist eine seltene Komplikation. Klinisch kann sie durch einen schmerzlosen Ikterus auffällig werden oder als Zufallsbefund auftreten. Bildgebend stellt sie sich in der Regel gut in der Sonografie dar. In der Literatur finden sich hierzu meistens Einzelfallberichte 305 306 307 308 309 310 311 312 . Eine retrospektive Studie untersuchte 135 Patienten und fand eine segmentale intrahepatische Cholestase in 4 (2,9 %) Fällen. Die meisten Fälle verlaufen mild und können klinisch und laborchemisch verlaufskontrolliert werden. Bei persistierend erhöhtem Serumbilirubin oder Cholangitis-Episoden zeigten sich biliäre Dekompressionen nach intern (via ERCP) oder extern (via PTCD) als effektiv 306 308 309 310 312 . In seltenen refraktären Fällen kann es zu nicht beherrschbaren Cholangitiden kommen, die eine Lebertransplantation erforderlich machen. Patienten mit klinisch apparenter mechanischer Cholestase nach TIPS sollten daher an einem Transplantationszentrum vorgestellt werden 305 307 310 .

#### Segmentale Leberischämien

Segmentale Leberischämien sind seltene prozedurale Komplikationen nach TIPS-Anlage. Klinisch kann sich ein abdominelles Druckgefühl, hepatische Enzephalopathie sowie später ein Ikterus präsentieren. Diagnostiziert wird die Leberischämie durch einen raschen Anstieg der Transaminasen (AST > ALT) sowie eine kontrastmittelverstärkte Bildgebung. Die verfügbare Literatur hierzu sind zumeist Fallberichte und Fallserien 313 314 315 316 317 318 319 320 . Die bislang größte retrospektive Analyse zeigte eine Rate von knapp 3 Prozent bildmorphologisch nachweisbarer segmentaler Perfusionsausfälle der Leber nach TIPS-Anlage 313 314 315 316 317 318 319 320 321 . Die meisten Fälle sind transient und ohne klinisch relevante Folgen für die Betroffenen 314 315 316 . Im Fall eines nicht transienten Verlaufs 313 317 319 320 321 sollte eine Lebertransplantation erwogen werden. 313 321 . Patienten mit (segmentaler) Leberischämie sollten daher an einem Transplantationszentrum vorgestellt werden.

### Ausblick

Der TIPS ist die effektivste Methode, den Pfortaderdruck zu senken und Komplikationen der Portalen Hypertension zu kontrollieren. Insbesondere in Deutschland kommt es zu einem zunehmenden und breiten Einsatz dieser Technik. Auch wenn nicht für alle Empfehlungen in dieser Arbeit hohe Evidenz mittels Analysen und randomisiert kontrollierten Studien gibt, sind diese Empfehlungen durch langjährige Expertise und multizentrische Zusammenarbeit und Erfahrungsaustausch belegt. Sicherlich muss noch mehr in diesem Bereich geforscht werden, um die Versorgung der Patienten, deren Auswahl und weitere Betreuung so optimal wie möglich zu gestalten.

## Figures and Tables

**Abb. 1:**
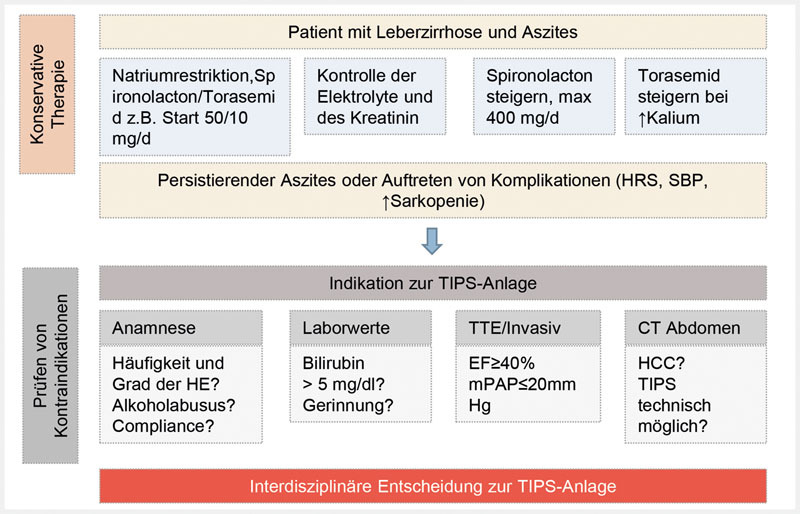
Flowchart Aszites. [rerif]

**Abb. 2:**
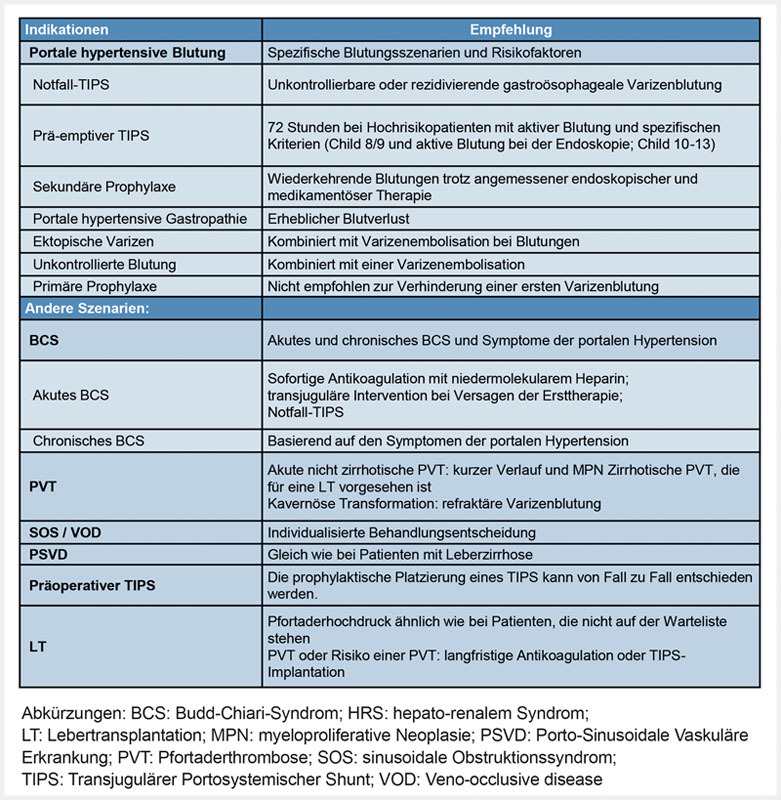
Indikation Blutung und weitere Szenarien. [rerif]

**Abb. 4:**
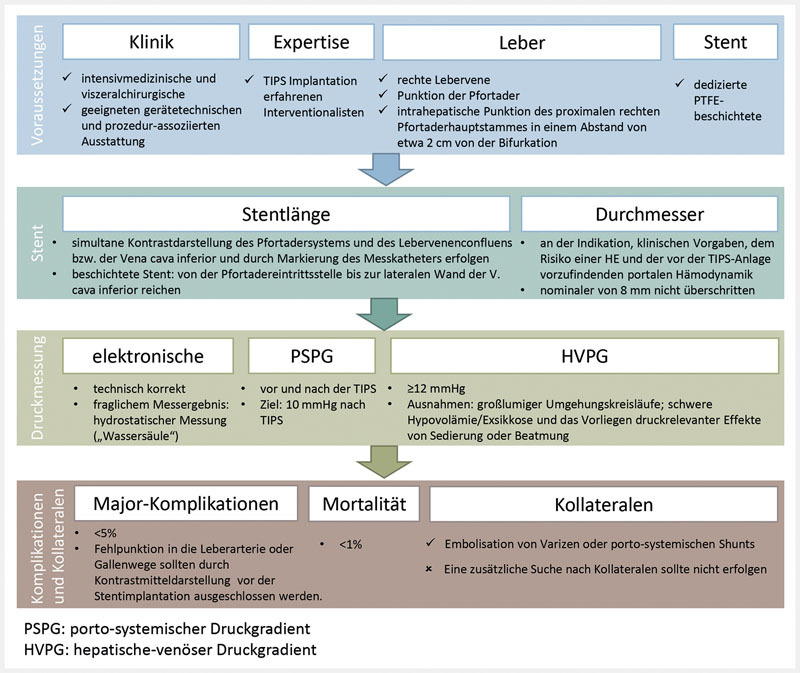
Technische Durchführung des TIPS. [rerif]

**Abb. 6:**
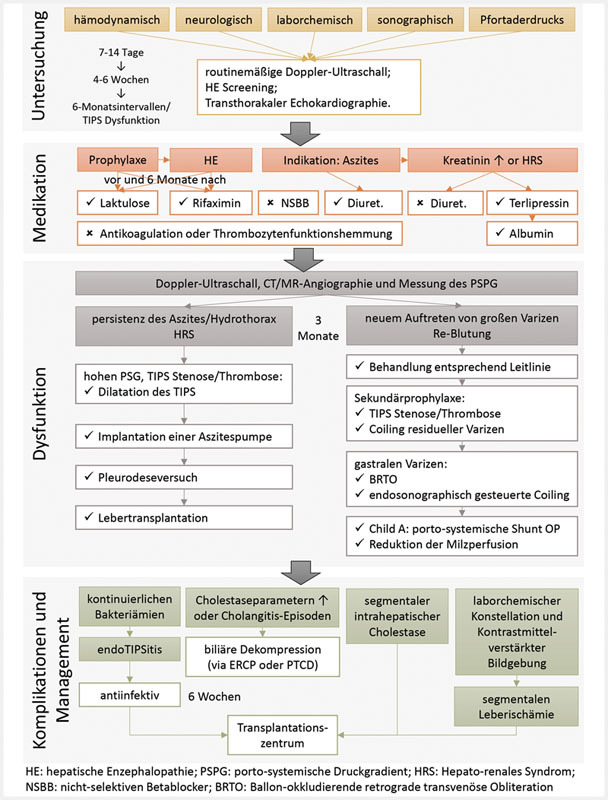
TIPS-Nachsorge. [rerif]

## Notes

Merkbox 1: TIPS bei Aszites und Nierenfunktionsstörung ( Abb. 1 )
Eine Evaluation zur TIPS-Anlage ist bei allen Patienten mit rezidivierendem und therapierefraktärem Aszites, und/oder hepatischem Hydrothorax und HRS-NAKI/CKD indiziert. Präinterventionelle Bestimmung der Routinelaborwerte (Leberschädigung und -synthese (Transaminasen, Cholestaseparameter, Albumin, Cholinesterase, Bilirubin), Nierenfunktion (Kreatinin), Gerinnung (INR) und Blutbild), NT-proBNP (s. Kapitel „kardiale Diagnostik“)-Bestimmung der Natriumausscheidung bei Patienten mit Aszites im Spontanurin für die Beurteilung der Diuretikarefraktärität, und Ausschluss einer strukturellen Nierenerkrankung (Mikroalbuminämie/Proteinurie). Ausschluss einer SBP (< 250 neutrophile Granulozyten/μl) durch eine diagnostische Parazentese bei Patienten mit Aszites. Bestimmung des Serum/Aszites Albumingradienten und/oder des Aszitesgesamteiweißes zum Ausschluss eines nicht-zirrhotischen Aszites. Präinterventionelle Parazentese/Thorakozentese bei Patienten mit Aszites oder Hydrothorax mit dem Ziel einer weitgehenden Entfernung von Aszites und/oder Pleurarerguss. Eine Lebertransplantation als kurative Therapie sollte bei allen Patienten grundsätzlich diskutiert werden.

## Notes

Merkbox 2: TIPS-Anlage bei portal-hypertensiver Blutung bei Leberzirrhose ( Abb. 2 )
**Primärprophylaxe:** keine routinemäßige TIPS-Implantation zur Verhinderung einer ersten Varizenblutung.
**Notfall-TIPS:** Notfall-TIPS-Implantation bei Patienten mit gastroösophagealer Varizenblutung, die nicht durch endoskopische und medikamentöse Maßnahmen zu kontrollieren ist, oder die innerhalb von 5 Tagen rezidiviert. Zeitpunkt: so schnell wie möglich.
**Präemptiver TIPS:** TIPS-Implantation bei Patienten mit Blutung aus gastroösophagealen Varizen und Child-Pugh B-Stadium (≥ 8) mit aktiver Blutung bei initialer Endoskopie oder Child-Pugh C-Stadium mit < 14 Punkten oder wenn ein HVPG > 20 mmHg bekannt ist. Zeitpunkt: möglichst innerhalb von 72 h nach Indexblutung.
**Sekundärprophylaxe:** TIPS Implantation, wenn bei Patienten trotz leitliniengerechter dualer endoskopischer und medikamentöser (nicht-selektive Betablocker) Therapie eine Rezidivblutung aus gastroösophagealen Varizen vorliegt. Ebenso ist bei vorliegenden Kontraindikationen einer dualen Prophylaxe der TIPS zu erwägen. Zeitpunkt: innerhalb von 2 Wochen nach Rezidivblutung.
**Portal-hypertensive Gastropathie:** Erwägen einer TIPS Implantation bei Patienten, die trotz leitliniengerechter Therapie mit nicht-selektiven Betablockern einen relevanten Blutverlust aus portal-hypertensiver Gastropathie erleiden.
**Ektope Varizen:** Erwägen einer TIPS Implantation kombiniert mit einer Embolisation der Varizen bei Blutungen aus ektopen (intestinalen) Varizen.
**Sondersituationen:** TIPS-Implantation zu erwägen (Einzelfallentscheidungen) bei endoskopisch und medikamentös nicht-durchführbarer Blutungsprophylaxe (z. B. ektope Varizen, Komorbiditäten) und bei unten aufgeführten weiteren Sondersituationen.

## Notes

Merkbox 3: TIPS-Anlage bei vaskulären Lebererkrankungen ( Abb. 2 )
**Budd-Chiari-Syndrom (BCS):**
 
Sofortige Antikoagulation mit niedermolekularem Heparin bei akutem BCS. Transjuguläre Intervention bei progredientem Leberversagen. Notfall-TIPS-Implantation bei akutem BCS mit fulminantem Leberversagen.
Indikation zur TIPS-Implantation bei chronischem BCS, ohne akute Kongestion, entsprechend der Symptome der portalen Hypertension (siehe **Merkbox 1 und 2** ).
**Akute Pfortader-Mesenterial- und Milzvenenthrombosen:**
 
Erwägen einer interventionellen Therapie (transjuguläre transhepatische Pfortaderlyse und TIPS-Anlage) bei der akuten nicht-zirrhotischen nicht-malignen Pfortaderthrombose (PVT) mit drohender Mesenterialischämie und/oder keiner Besserung unter der Antikoagulation im kurzfristigen Verlauf (max. 2 Wochen). Bei Vorliegen eines malignen Thrombus ist ein TIPS nicht sinnvoll. Die zirrhotische PVT allein ist per se keine Indikation zur TIPS-Anlage. Erwägen einer TIPS-Implantation bei Komplikation der portalen Hypertension, bei Progress unter Antikoagulation, und insbesondere wenn Patienten für eine Lebertransplantation vorgesehen sind. **Portalvenöses Kavernom:**
 
Erwägen einer TIPS-Implantation, falls technisch möglich, bei kavernöser Transformation und Indikationsstellung analog zu Patienten mit Leberzirrhose sowie bei portal-kavernöser Cholangiopathie. **Sinusoidales Obstruktionssyndrom:**
 
Erwägen einer TIPS-Implantation bei sinusoidalem Obstruktionssyndrom (SOS) als individuelle Therapieentscheidung der Komplikationen der portalen Hypertension. **Porto-Sinusoidale Vaskuläre Erkrankung (PSVD):**
 
Indikationsstellung zur TIPS-Anlage bei der PSVD entsprechend der Indikation zur TIPS -Anlage bei Patienten mit Leberzirrhose.

## Notes

Merkbox 4: TIPS-Anlage vor Operation und Transplantation Prophylaktische TIPS-Anlage vor abdominal-viszeralchirurgischen Operationen mit klinisch signifikanter portaler Hypertension erfolgt auf Basis einer Einzelfallentscheidung. Indikationsstellung für eine TIPS-Implantation bei Patienten auf der Transplantationswarteliste in Analogie zu Patienten mit portaler Hypertension, die nicht auf der Warteliste sind. Bei Patienten mit PVT oder einem hohem Risiko einer PVT ist eine Dauerantikoagulation und/oder eine TIPS-Implantation zu erwägen. Berücksichtigen der Notwendigkeit einer Transplantation durch optimale Platzierung des Stents (siehe Merkbox 6) bei der TIPS-Implantation.

## Notes

Merkbox 5: Patientenselektion und Kontraindikationen TIPS-Implantation ist mit erhöhtem postinterventionellem Komplikationsrisiko bei Patienten mit einem sehr hohem BMI (> 45) oder ausgeprägter Kachexie (BMI < 18,5) verbunden. TIPS-Implantation ist bei erwachsenen Patienten jeden Alters möglich, bei älteren Patienten jedoch mit erhöhtem Risiko für die Entwicklung einer Hepatischen Enzephalopathie. Die TIPS-Implantation sollte möglichst in einem Infekt-freien Intervall oder erst nach kontrollierter Infektion stattfinden. Eine aktive und insbesondere unkontrollierte Infektion oder Sepsis stellt eine Kontraindikation für die TIPS-Implantation dar. Durchführung einer kardialen Diagnostik mit 12-Kanal-EKG, einer transthorakalen Echokardiografie und der Bestimmung des NT-proBNP vor TIPS-Implantation.
Toulouse-Algorithmus: Bei NT-proBNP < 125 pg/ml ist die TIPS-Implantation sicher, bei NT-proBNP ≥ 125 pg/ml aber E/A < 1.5, E/e' < 10 und linksatrialer Volumensindex (LAVI) < 34 ml/m ^2^ ein niedriges Risiko und bei E/A > 1.5 oder E/e' > 10 oder LAVI > 34 ml/m ^2^ ein hohes Risiko einer kardialen Dekompensation nach TIPS zu erwarten.
Auch eine linksventrikuläre Ejektionsfraktion < 40 % und eine pulmonal-arterielle Hypertonie mit einem mittleren pulmonal-arteriellen Druck von > 30 mmHg gelten als Kontraindikationen für eine TIPS-Anlage. Zirrhotische Kardiomyopathie mit linksventrikulärer diastolischer Funktionsstörung sowie höhergradige Aortenklappenstenosen gelten als Risikofaktoren für kardiale Dekompensation nach TIPS-Anlage. Durchführung einer Duplex-Ultraschalluntersuchung des Oberbauchs zur Beurteilung des Leberparenchyms, der Pfortader, der Leberarterie und des T. coeliacus, außerdem Beurteilung der Menge des Aszites und des Hydrothoraxes im Rahmen der Planung einer TIPS-Implantation. Durchführung eines kontrastmittelverstärkten Mehrphasen-CT oder -MRT natives Leber-MRT der Oberbauchorgane, im Rahmen der Planung einer TIPS-Implantation.
Routinelaborparameter der Blutgerinnung bei Zirrhose sind von untergeordneter Bedeutung. Aus der Anamnese ableitbare Blutungsneigung sind entscheidend über das Blutungsrisiko, vergleichbar mit anderen interventionellen Eingriffen (TZ ≤ 50 000/mm ^3^ und INR ≥ 2,5), ohne regelhafte prophylaktische Gabe von Thrombozyten oder Gerinnungsfaktoren.
TIPS-Implantation zur Aszitestherapie bei vorbestehender chronischer hepatischer Enzephalopathie ≥ Grad 2 (ohne Auslöser) oder einem Serum-Bilirubin > 5 mg/dl ist in der Regel kontraindiziert. TIPS-Implantation bei Patienten mit HCC und therapierefraktärem Aszites im Rahmen individualisierter Therapiekonzepte nach interdisziplinärer Abstimmung (nicht durch den Tumor, nicht bei Peritonealkarzinose oder Pfortaderinvasion). Vorhandensein eines TIPS ist keine absolute Kontraindikation gegen die (superselektive) transarterielle Chemoembolisation von HCCs, jedoch Vorsicht ist geboten.

## Notes

Merkbox 6: Technische Aspekte einer TIPS-Anlage ( Abb. 4 )
Vorhandensein einer geeigneten technischen Ausstattung, sowie erfahrenes (min. 20 TIPS/Jahr) interdisziplinäres Team (interventionell-radiologische, internistisch-interventionelle, Intensiv-medizinische und viszeralchirurgische Expertise) in der Behandlung von Patienten mit dekompensierter Leberzirrhose. Rate an Major-Komplikationen bei der TIPS-Neuanlage unter 5 %, die Letalitätsrate unter 1 %. Verwenden der rechten Lebervene als Standardzugang bei erhaltener Anatomie. Durchführung einer Sonografie mit farbkodierter Duplexsonografie unmittelbar vor der TIPS-Anlage. Pfortaderpunktion mittels perkutaner Ultraschall-(US)-Führung zur Senkung der Strahlendosis und Reduzierung von Komplikationsraten und Eingriffszeit. Bevorzugt intrahepatische Punktion des proximalen rechten Pfortaderhauptstammes in einem Abstand von etwa 2 cm von der Bifurkation. Ausschluss von Fehlpunktion der Leberarterie oder Gallenwege durch Kontrastmitteldarstellung vor der Stentimplantation. Verwendung von PTFE-beschichteten TIPS-Stentgrafts. Ermittlung der Stentlänge durch simultane Kontrastdarstellung des Pfortadersystems und des Lebervenenconfluens bzw. der Vena cava inferior und durch Markierung des Messkatheters. Länge zur Positionierung des gecoverten Stents von der Pfortadereintrittsstelle bis kurz vor oder zur Mündung in die V. cava inferior. Technisch korrekte Durchführung der digitalen Druckmessung. Überprüfung mittels hydrostatischer Messung („Wassersäule“) bei fraglichem Messergebnis. Ermitteln des Druckgradienten vor und nach der TIPS-Anlage aus dem Druck in der Pfortader und jenem in der Vena cava inferior am Eintritt der Lebervenen, hieraus ergibt sich der porto-systemische Druckgradient (PSPG). Messung des PSPG oder HVPG (Lebervenenverschlussdruck) vor der Stent-Implantation (Gradient ≥ 10 mmHg stützt die Indikation für eine TIPS-Anlage). In der Regel keine TIPS-Implantation bei Patienten mit einem PSPG < 10 mmHg. Ausnahmen können u. a. sein: der Nachweis großlumiger Umgehungskreisläufe (z. B. spleno-renaler Shunt), eine schwere Hypovolämie/Exsikkose, präsinusoidale portale Hypertension (z. B. PSVD) und das Vorliegen druckrelevanter Effekte von Sedierung oder Beatmung. Ziel-PSPG nach TIPS-Implantation ist ≤ 12 mmHg bei Indikation Blutung und eine > 60 % Senkung des PSPG bei Indikation Aszites. Auswahl des Stentgraftdurchmessers orientierend an der Indikation (Varizenblutung, Aszites, Budd-Chiari-Syndrom), den klinischen Vorgaben (Alter, Bilirubinkonzentration), dem Risiko einer hepatischen Enzephalopathie und der vor der TIPS-Anlage vorzufindenden portalen Hämodynamik (prograder vs stagnierender/retrograder Fluss). Meist ist ein effektiver Stentgraftdurchmesser von 8 mm bei TIPS-Neuanlage notwendig bzw. ausreichend. Durchführung einer (adjuvanten) Embolisation bei großen Varizen trotz niedrigem PSPG (< 15 mmHg) und Erwägen einer Embolisation von Varizen oder porto-systemischen Shunts bei Indikation Blutung, bei bestehender Kollateralperfusion trotz TIPS oder unzureichender PSPG-Senkung.

## Notes

Merkbox 7: Postinterventionelles Management Postinterventionelle hämodynamische, neurologische und laborchemische Überwachung nach TIPS-Implantation, je nach Risikostratifizierung (ggf. IMC/ITS). Routinemäßige Doppler- und Ultraschall-Untersuchung 1–7 Tage postinterventionell (vor Entlassung) zum Ausschluss von Blutung, Hämatom und TIPS-Funktion, danach in 4–6 Wochen, dann in 3 Monaten und schließlich in 6-Monatsintervallen (Screening für HCC) und bei jedem Verdacht auf eine TIPS-Dysfunktion. Angiografische Darstellung des TIPS inklusive Druckmessung, im Fall einer sonografisch vermutetem TIPS-Dysfunktion, bei unklaren sonografischen Befunden oder fehlendem klinischen Erfolg (portal-hypertensiver Komplikationen). Screening auf klinisch-manifeste HE anhand der West-Haven-Kriterien im Rahmen der regelmäßigen Nachsorge. Durchführung einer transthorakalen Echokardiografie bei klinischem Verdacht auf eine kardiale Dekompensation. Aktuell aufgrund der limitierten Evidenz keine Empfehlung für eine spezifische Antikoagulation oder Thrombozytenfunktionshemmung nach TIPS-Anlage, außer bei prothrombotischen Ereignissen. Beginn mit Rifaximin vor der TIPS-Implantation und Fortsetzung für 6 Monate nach TIPS-Anlage bei Patienten mit früheren klinisch-manifesten HE-Episoden. Diuretikatherapie mit rascher Dosiserhöhung bei der Indikation Aszites und normwertigem Kreatinin. Vorsichtiger Beginn bei erhöhtem Kreatinin oder bei HRS.

## Notes

Merkbox 8: Management der TIPS-Dysfunktion ( Abb. 6 )
Invasive TIPS-Darstellung beim erneuten Auftreten von Aszites, eines hepatorenalen Syndroms, großer Varizen oder einer portal-hypertensiven Blutung nach TIPS-Anlage. Diagnostik in der Sequenz Doppler-Ultraschall, CT/MR-Angiografie und Messung des porto-systemischen Druckgradienten. Dilatation/Stenting des TIPS bei Nachweis eines hohen Druckgradienten bzw. TIPS-Stenose/Thrombose. TIPS-Revision und gegebenenfalls in gleicher Sitzung Coiling von noch vorhandenen Restvarizen. Erwägen des sogenannten Ballon-okkludierenden retrograden transvenösen Verschlusses (BRTO) oder das endosonografisch gesteuerten Coilings der Varizen (+/– Histoacryl) als alternative Therapieoptionen bei gastralen Varizen. Evaluation der Möglichkeit einer porto-systemischen Shunt-OP bei Patienten im Stadium Child A mit rezidivierenden Blutungen nach TIPS-Anlage und ohne Option der Verbesserung der TIPS-Funktion (oder eines Parallel-TIPS) in einem in der Shuntoperation erfahrenen Zentrum. Erwägen einer Reduktion der Milzperfusion zur Pfortaderdrucksenkung, und einer Sperr-Operation als Ultima Ratio. Prüfen der Möglichkeit einer Lebertransplantation bei allen Patienten mit TIPS-Dysfunktion und fehlender interventioneller Möglichkeit der Verbesserung der TIPS-Funktion. Erwägen der Implantation einer Aszitespumpe oder getunnelte Katheter bei Persistenz einer hohen Parazentesefrequenz mit deutlicher Beeinträchtigung der Lebensqualität, ohne SBP in der Vorgeschichte und fehlender Option der Verbesserung der TIPS-Funktion (erfahrene Zentren). Versuch der Pleurodese bei Persistenz eines hepatischen Hydrothorax trotz o. g. Therapieoptionen.

## Notes

Merkbox 9: Management der TIPS-Komplikationen Erwägen einer EndoTIPSitis bei klinisch signifikanten kontinuierlichen Bakteriämien (Fieber und mehrere positive Blutkulturen) mit Vegetationen oder Thrombus innerhalb des TIPS als Differenzialdiagnose. Vorliegen einer EndoTIPSitis auch bei Patienten mit durchgängigem TIPS nach Ausschluss anderweitiger Infektionsquellen und bei anhaltender Bakteriämie und persistierendem Fieber möglich. Behandlung einer EndoTIPSitis intravenös, antiinfektiv über mind. 6 Wochen. Vorstellung von Patienten mit EndoTIPSitis an einem Transplantationszentrum. Diagnose der segmentalen mechanischen Cholestase mittels Bildgebung (z. B. Ultraschall). Erwägen einer biliären Dekompression (via ERCP oder PTCD) bei persistierend erhöhten laborchemischen Cholestaseparametern oder Cholangitis-Episoden. Vorstellung von Patienten bei komplikativer persistierender (segmentaler) mechanischer Cholestase an einem Transplantationszentrum. Diagnose der (segmentalen) Leberischämie anhand entsprechender laborchemischer Konstellation und kontrastmittelverstärkter Bildgebung. Bei Zeichen des beginnenden Leberversagens Vorstellung an einem Transplantationszentrum.
